# The process of co‐designing a model of social prescribing: An Australian case study

**DOI:** 10.1111/hex.14087

**Published:** 2024-05-24

**Authors:** Candice Oster, Ashleigh Powell, Claire Hutchinson, Debra Anderson, Bill Gransbury, Martin Walton, Jenny O'Brien, Susan Raven, Svetlana Bogomolova

**Affiliations:** ^1^ Caring Futures Institute, College of Nursing and Health Sciences Flinders University Adelaide South Australia Australia; ^2^ Centre for Social Impact, College of Business, Government and Law Flinders University Adelaide South Australia Australia

**Keywords:** co‐design, health services, methods, social prescribing, social services

## Abstract

**Introduction:**

Social needs such as housing, employment, food, income and social isolation are having a significant impact on individuals, families and communities. Individuals are increasingly presenting to health settings with social needs, which are ill‐equipped to address nonmedical needs. Social prescribing is a systematic approach connecting the health, social and community sectors to better address social needs and improve health and wellbeing. Social prescribing interventions are being implemented world‐wide. With variability in health and social care systems internationally, it is important that social prescribing interventions are co‐designed with key stakeholders to ensure they can be implemented and sustained within local systems.

**Methods:**

This Australian case study provides a detailed description of the process undertaken to co‐design a social prescribing service model in a regional area. Four co‐design workshops were undertaken, two with health and social care professionals and two with community members. The project followed an iterative process of resourcing, planning, recruiting, sensitising, facilitation, reflection and building for change across the workshops.

**Results:**

Through this process, key stakeholders were able to successfully co‐design a social prescribing model of care for the region.

**Conclusion:**

By demonstrating the process and materials used in our project, we aim to open the ‘black box’ of co‐design for social prescribing and provide ideas and resources for others to adapt and utilise.

**Patient or Public Contribution:**

The project was designed and undertaken by a steering committee comprising university‐based researchers (authors C. O. and S. B.), local government (author D. A.) and health, social and community services (authors B. G., M. W., J. O. and S. R.). Members of the steering committee participated in project design, participant recruitment, workshop facilitation, data analysis and interpretation.

## 1 INTRODUCTION

Social prescribing is a systematic approach in health and community settings to refer people to social activities and social services.^1^, ^2^ It is a response to the growing recognition of the effects of nonmedical issues such as a lack of housing, employment, food, income and social inclusion on individuals, families and communities. Social prescribing leverages the support provided by social and community services to address nonmedical needs and improve health, wellbeing and social connection.^2^ Social prescribing is often delivered through health settings due to the frequency of visits related to nonmedical needs. For example, in Australia the Royal Australian College of General Practitioners recently reported that up to 36% of patient presentations to general practitioners (GPs) are due to the effects of nonmedical issues on health.^3^

Social prescribing is a heterogeneous concept, demonstrated in a recent scoping review of social prescribing programmes.^4^ The study found that social prescribing programmes differ regarding the implementation context, population of focus and nonmedical needs addressed. Further differences were observed in how these programmes are staffed, the degree of support and follow‐up provided to programme participants and referral pathways and processes. Models of social prescribing can be as simple as providing information to individuals about the services that are available (termed ‘signposting’) to more holistic models. Holistic models involve a ‘link worker’ meeting with the person to identify their nonmedical needs, actively supporting them to access services, providing care planning, motivation and goal setting interventions, and following up over weeks or months.^4^

There is a long history of social prescribing in the United Kingdom, with the National Health Service's long‐term plan^5^ aiming for at least 900,000 people to be referred to social prescribing by 2023/2024. There is also increasing uptake of social prescribing worldwide.^2^ There is emerging literature that examines the effectiveness of various social prescribing models.^6^, ^7^ However, because countries differ in the extent to which they fund and invest in health and social care and the degree of integration between health and social care systems,^8^ there is variation in the ways in which social prescribing is embedded in these systems.^4^ This means that international models of social prescribing cannot be transposed into different geographic and systemic contexts. It is important, therefore, that social prescribing models be designed with input from key stakeholders, including consumers and health and social care providers. Further, social prescribing models need to be informed by context to ensure the programme is ‘fit’ for the systems in which they will be enacted.

Accordingly, there is support for social prescribing to be *co‐designed* with key stakeholders.^1^, ^9^ Co‐design is an evolving concept that means different things to different people.^10^, ^11^ Due to this ‘conceptual fuzziness’,^10^ ^,p.1578^ it is recommended that researchers clarify their meaning of the term. Here, we define co‐design as ‘… active collaboration between stakeholders in designing solutions to a prespecified problem’.^12^ ^,p.2^

Co‐design involves more than consulting key stakeholders about service design. It is a collaborative process of engagement and decision‐making where key stakeholders are empowered to make decisions about service design that is reflective of their experiences and perceptions. This is commonly done through co‐design workshops, where key stakeholders come together to collectively design a solution to a shared problem. While the relevance of co‐design is clear, there is little detailed information on the *process* of co‐designing a social prescribing model of care. For example, existing guidance, such as the World Health Organization's social prescribing toolkit,^13^ provides valuable information on how to implement social prescribing but does not detail the process of designing the programme in the first instance.

Social prescribing has yet to be widely adopted in Australia. However, it is included in Australia's National Preventive Health Strategy 2021–2030^14^ and Primary Health Care 10 Year Plan 2022–2032.^15^ With a growing interest in social prescribing in Australia, the objective of this case study is to demonstrate the co‐design process undertaken to inform a social prescribing service model in a regional area of Australia. The process outlined involves stakeholder identification and mapping, materials used for sensitising participants and guiding the discussions, steps undertaken, analysis approach and outputs and outcomes generated. In addition to providing preliminary findings regarding social prescribing service models, the purpose of this case study is to provide others with a clear method of using co‐design for social prescribing that could be adapted and applied in other contexts.

## 2 MATERIALS AND METHODS

We followed Trischeler et al.'s^16^ seven step co‐design process, described in Table 1. The process was designed to empower participants during co‐design.^17^ It provided comprehensive guidance on co‐design steps and the use of co‐design tools to engage key stakeholders and ensure their voices, experiences and needs remained central to the co‐design process.^18^ The application of the seven co‐design steps in the case study are detailed below.

**Table 1:** The seven‐step co‐design process.

Step	Description
(1) Resourcing	Gain an initial understanding of the problem/task to be addressed (e.g., through literature reviews, interviews, surveys)
(2) Planning	Work with key stakeholders to determine the design task (goals and outcomes) and plan the next stages of co‐design
(3) Recruiting	Systematically identify, screen and recruit suitable participants
(4) Sensitising	Prepare participants for the design task and trigger reflections on the topic through activities such as presentations and thought‐provoking questions
(5) Facilitation	Use co‐design tools to foster creativity in individual activities and group discussion (e.g., card sorting)
(6) Reflecting	Reflect on the co‐design outcomes
(7) Building for change	Open dialogue with key stakeholders to assess feasibility and realisation of the ideas generated in the workshop(s)

*Note*: The table is adapted from Trischeler et al.^16^

The project was granted approval by the Flinders University Human Research Ethics Committee ethics committee (Project Number 4868).

### 2.1 Setting

The project was conducted in a regional area of Australia where healthcare providers and local government were interested in developing social prescribing for the region (Note: in Australia the term ‘regional’ designates towns and small cities outside of the major capitals). The population size was estimated at 25,869 with around 29 persons per square kilometre. In 2021, 12,264 people in the region were employed (58% full‐time; 35% part‐time). Consistent with the ageing population in Australia, in 2021 those aged 60–64 years old were the largest age group (data from https://profile.id.com.au/barossa/).

Like many areas in Australia, the region's population is experiencing the negative effects of a range of factors, including the rising cost of living, homelessness and social isolation.^19^ Participants in this co‐design study told us that health and social care providers in the region historically work in ‘silos’, with no formal referral pathways between sectors. Healthcare providers in our study further reported frequently seeing clients with nonmedical (social) needs and reported difficulties in helping their clients address these needs. They described consequent effects as ‘clinician burnout’, ‘vicarious trauma’ and ‘compassion fatigue’. The project aimed to co‐design a social prescribing model of care for the region to better connect the health, social care and community sectors to support community members experiencing social needs.

### 2.2 Co‐design workshops

As discussed, social prescribing is a complex concept, with multiple components. These components can be brought together in different ways depending on the population or issue of focus, and contextual factors regarding how health and social care systems function. The aim of the workshops was to draw on the experiences of health and social service providers as well as community members to understand what components of social prescribing would be applicable to the region and how these could be brought together into a model of care.

Four co‐design workshops were conducted between July and November 2023. Two were run with health and social service providers (*n* = 19 in Workshop 1, *n* = 16 in Workshop 2). Recruitment was done via stakeholder mapping and leveraging steering committee member networks (discussed below). Further, two co‐design workshops were run with community members. The first community member workshop involved participants recruited through a retirement village and aged care facility (*n* = 13). Participants in the second community workshop were recruited via flyers, advertisements and social media (*n* = 24).

Participant demographics are presented in Tables 2 and 3. Workshops were held in various locations in the region, typically in a hotel function room setting. Workshops lasted approximately 90 min. Participants in service provider workshops were provided a two‐course meal before the workshop. Community member participants were provided food and beverages and a $50 gift voucher each.

**Table 2:** Workshop participant demographics (service provider workshops).

Demographic	Workshop 1 (*n* = 19)^a^	Workshop 2 (*n* = 16)^a^
Profession		
Allied health	8	7
GP	2	2
Social service provider	9	7
Year in profession		
1 year or less	2	0
2–5 years	4	4
>5 years	12	11
Gender		
Male	1	3
Female	17	11
Age		
Under 25 years old	0	0
26–35 years old	4	2
36–45 years old	4	3
46–55 years old	7	5
56–64 years old	1	3
65+ years old	2	2

Abbreviation: GP, general practitioner.

a Some participants did not provide full demographic data.

**Table 3:** Workshop participant demographics (community member workshops).

Demographic	Workshop 1 (*n* = 13)	Workshop 2 (*n* = 24)
Gender		
Male	5	6
Female	8	18
Age		
Under 25 years old	0	0
26–35 years old	0	0
36–45 years old	0	4
46–55 years old	0	3
56–64 years old	0	9
65+ years old	13	8
Time living in the region^a^		
1 year or less	1	1
2–5 years	3	4
>5 years	8	19

a This information was of interest to the research team given that social prescribing aims to connect people to resources in the community and time in the region may be one factor related to social connectedness.

### 2.3 Co‐design steps and materials

The co‐design steps (resourcing, planning, recruiting, sensitising, facilitation, reflection and building for change) and materials used in each workshop are described below. Materials used for co‐design are available as Supporting Information S1: Data.

#### 2.3.1 Resourcing

Resourcing is a critical step to gain an understanding of problem being addressed through co‐design, ensuring that the ‘problem space is open to alternative solution spaces’,^16^ ^,p.1609^ rather than attempting to narrow it down to the expert‐driven solutions. This is often done through literature reviews, surveys, and interviews.

**Figure 1:**
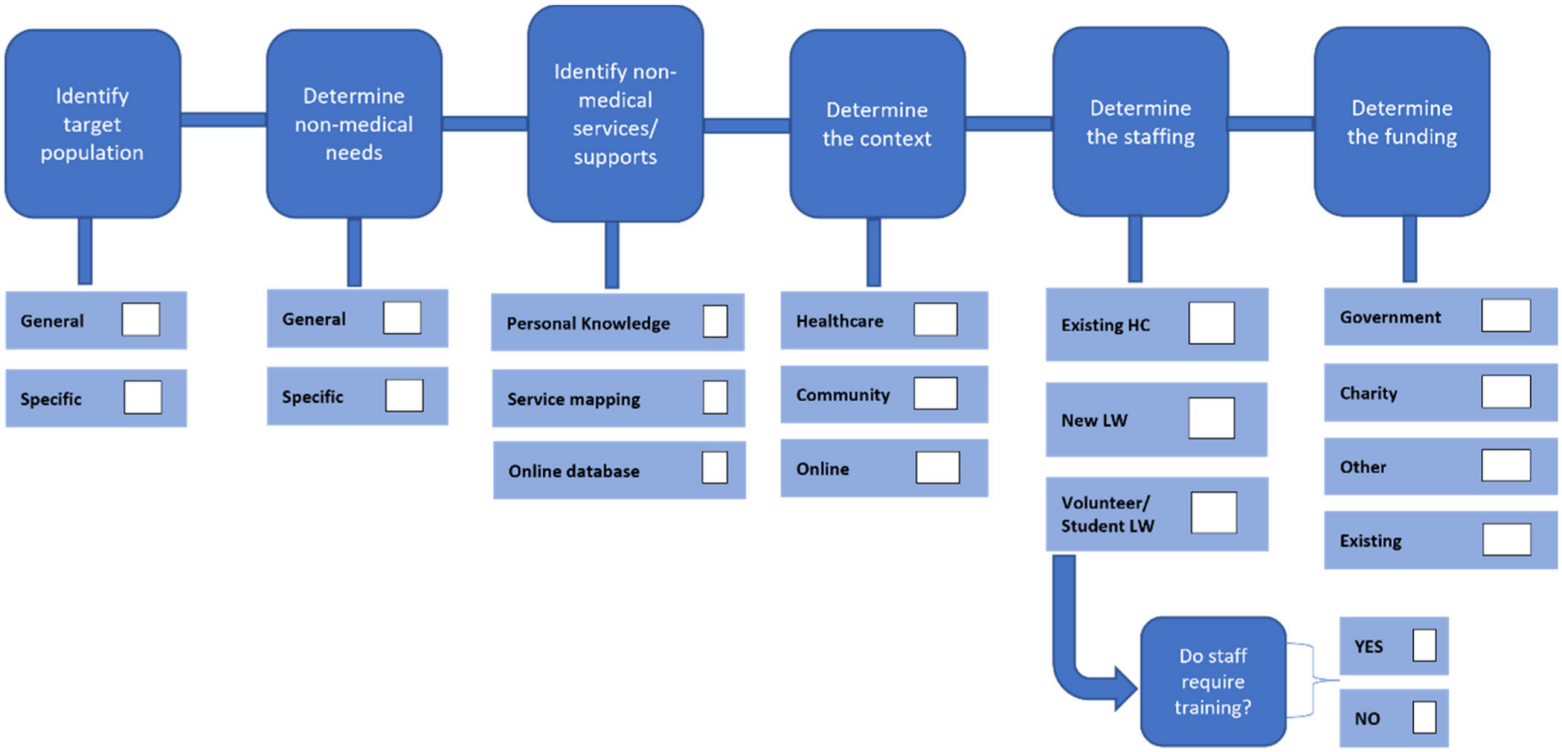
Social prescribing planning stages. HC, health care; LW, link worker.

**Figure 2:**
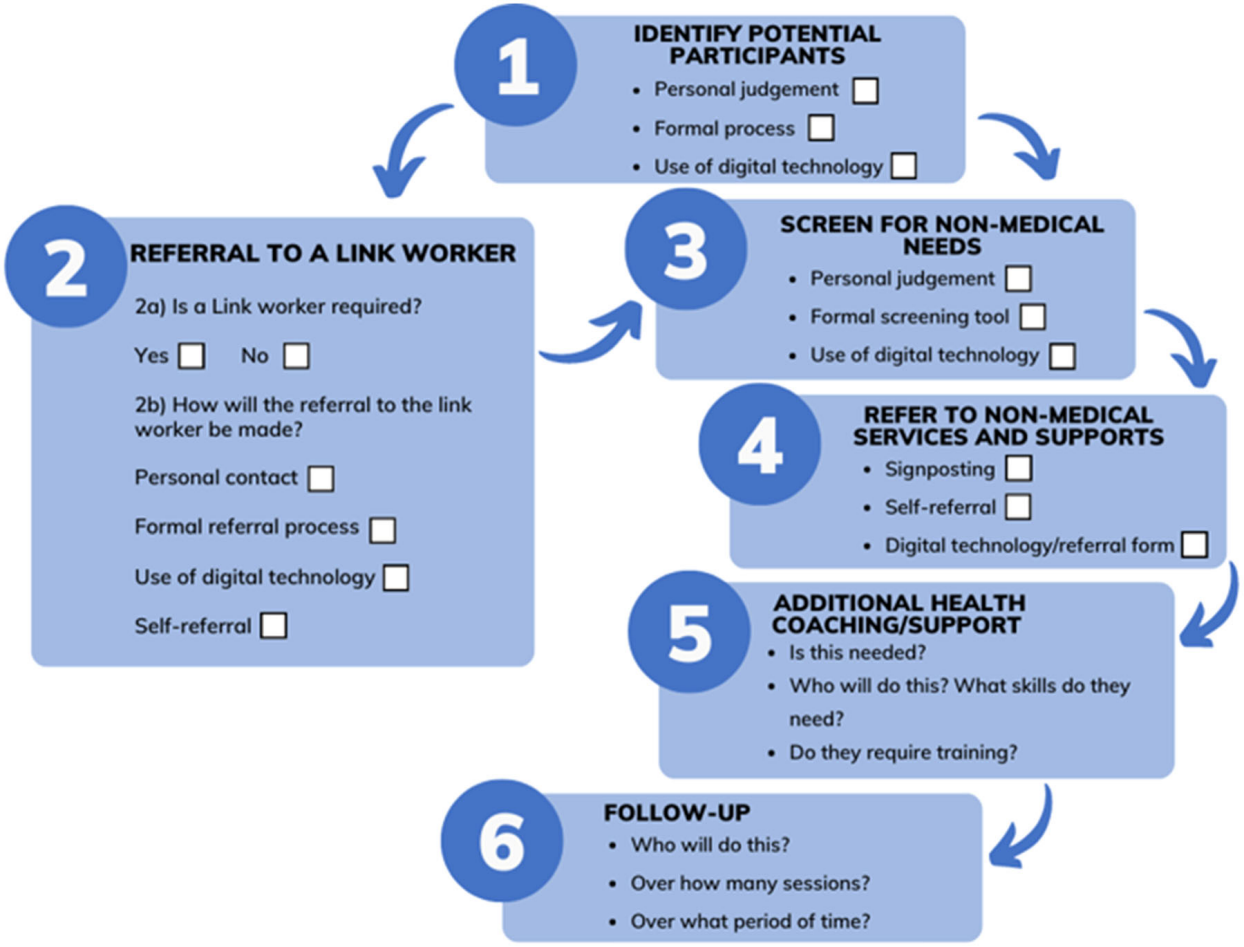
Social prescribing process stages.

We began the resourcing phase for the first workshop by undertaking a scoping review of components and models of social prescribing in the international literature. From this we determined six planning and six process stages for decision‐making during social prescribing co‐design^4^ (see Figures 1 and 2).

**Figure 3:**
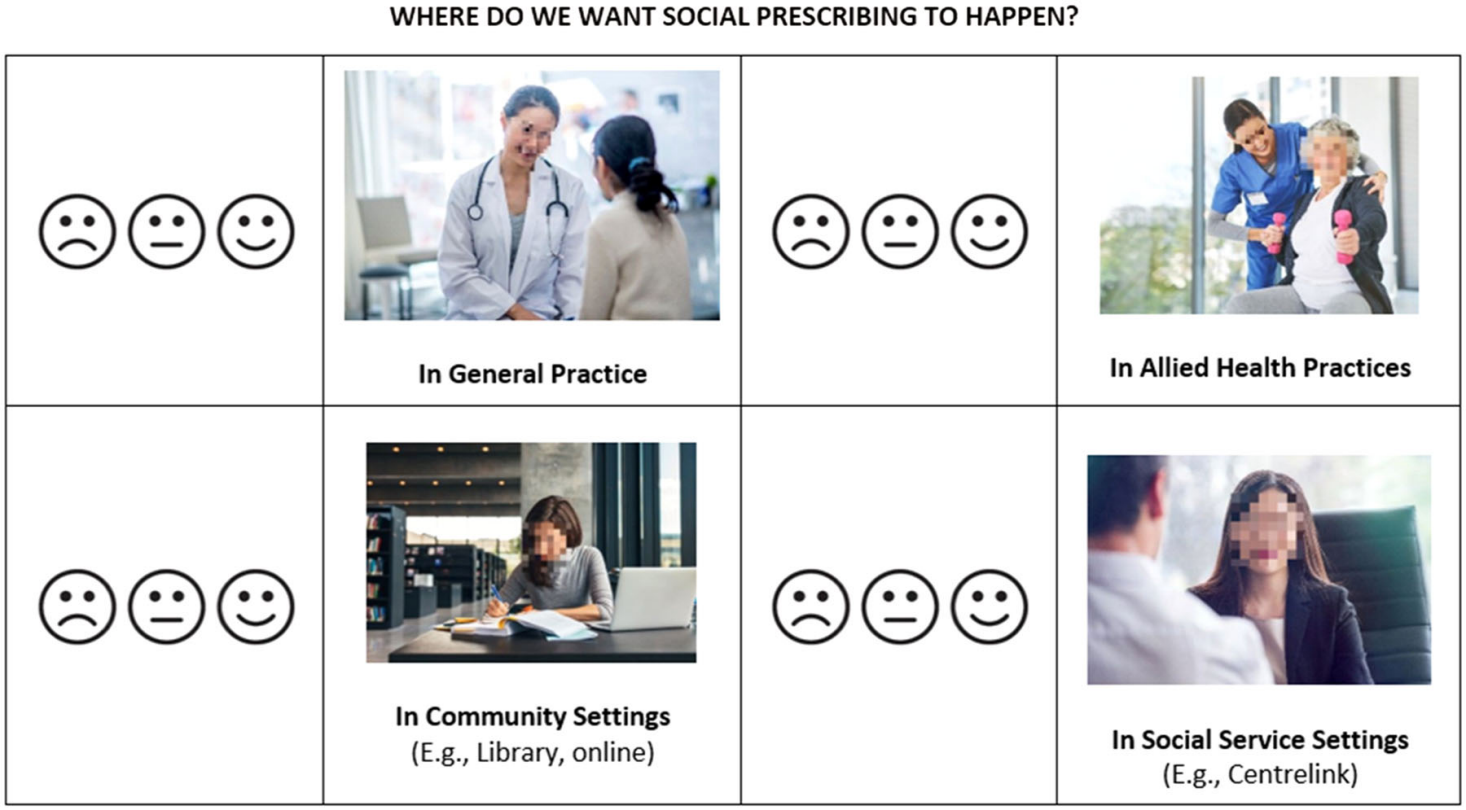
Ideas Workbook example page (note: images are stock images from Microsoft Word, masked as per journal guidelines).

The components of social prescribing across the planning and process stages were incorporated into an Ideas Workbook (see Supporting Information S1: Data), which allowed workshop participants to rate their feelings about different ideas (like, neutral, dislike) regarding the various components of social prescribing. Workbooks were completed by participants during the first service provider workshop (discussed further below). Figure 3 shows an example page of the Ideas Workbook.

We also conducted a community needs assessment to explore the nonmedical needs experienced by the community. This involved community members (*n* = 602) completing a validated social needs survey^20^ via door‐to‐door recruitment and intercepts in public locations (e.g., markets, sporting clubs, shopping centres). Survey data were analysed descriptively to explore the social needs experienced by the community. The average social needs score was 6.53 (scored on a 0–8 scale, with higher scores indicating fewer social needs). In terms of determining low, moderate and high need, we classified those who scored 0–3 on the social needs measure as high need, those who scored >3–6 as moderate need, and those who scored >6 as low need. Results identified that most participants experienced a low (64%) to moderate (35%) social needs and 1% experienced high levels of need. The community needs assessment was presented to participants at the start of the first workshop to provide an overview of social needs in the community.

Additionally, four focus groups (FG) were undertaken, two with community members (FG1 *n* = 10; FG2 *n* = 7) and two with health providers (FG1 *n* = 10; FG2 *n* = 6). FGs explored participant views on support for nonmedical needs and social prescribing. Health providers (*n* = 36) also completed a survey to explore their attitudes to social prescribing.^21^ FG and survey data were analysed descriptively (this data was reported as part of the South Australian Healthy Towns Challenge grant and is not reported here).

A final element of resourcing was setting up a steering committee to guide the project, which included the researchers plus representatives of the local council, general practice, allied health, social care and community groups. The steering committee was an important element in guiding all elements of the project and was integral to identifying and recruiting relevant stakeholders across siloed systems to participate in workshops.

#### 2.3.2 Planning

Planning was an iterative process^16^ with the steering committee meeting regularly over the course of the project. Early meetings involved developing the theory of change (explanation of how and why project activities aim to achieve project outcomes) and project logic (description of project inputs, activities, outputs, and outcomes) for the project (see Supporting Information S1: Data) and planning the workshop format.

Results from community FGs during the resourcing phase indicated a reliance on family and social media for support for nonmedical needs, and little knowledge or understanding of the concept of social prescribing and how health providers could be involved in supporting people with their nonmedical needs. Health providers demonstrated positive attitudes towards social prescribing and identified the need for a social prescribing programme in the region. Given the lack of knowledge of social prescribing on the part of community members and the need to ensure the co‐designed model of care would fit with existing practices across health and social care services, the decision was made to hold separate workshops with service providers and community.

We commenced with two workshops with service providers to begin determining which components of social prescribing would be included and how these could feasibly come together into a model of care. Two community workshops were then planned for community members to input into what they would like to happen at each stage of the model (termed the ‘social prescribing client journey’; see below). Community members could also discard aspects of the model and propose new ones. Regular steering committee meetings were held to reflect on each workshop and plan for the next.

#### 2.3.3 Recruiting

Recruiting was also an iterative process. Before each workshop, the steering committee conducted key stakeholder mapping to determine who to invite for the subsequent workshop. Recruitment was facilitated by steering committee members, who disseminated advertisements for the project through their channels (local newspapers, social media, flyers, networks). From this point in the co‐design process, the steps were somewhat different for service provider workshops and those with community members, as described below.

#### 2.3.4 Sensitising, facilitation and reflecting

In what follows, we describe the sensitising, facilitation, and reflecting across the four workshops.

Sensitising is ‘aimed at engaging potential participants and triggering reflection on the underlying topic prior to co‐design facilitation’.^16^ ^,p.1609^ This step is critical to provide participants an understanding of the problem space and the confidence to develop their own ideas.^16^ Sensitising was important for this co‐design project because social prescribing is a relatively new term and concept in Australia and is a complex process.

Facilitation involved the use of a range of design tools to empower participants and facilitate collaboration during co‐design.^17^ Design tools are ‘tools for conversation’, such as posters, slideshows, videos and possibility cards.^17^ ^,p.667^ Reflecting was undertaken through ongoing steering committee meetings to reflect on workshops, plan for further workshops and discuss and explore feasibility and realisation of the proposed model of care.

### 2.4 Service Provider Workshop 1: Co‐designing a draft social prescribing model of care

The scoping review and needs analysis formed the basis of sensitising in the first health and social service provider workshop. This included a PowerPoint presentation where the concept of social prescribing was described (including a video describing social prescribing from the United Kingdom; https://www.youtube.com/watch?v=O9azfXNcqD8), the scoping review results and needs analysis data were presented, and the co‐design process explained. Participants also responded individually to the ideas presented in the Ideas Workbook, indicating their likes/dislikes of the various components of social prescribing.

**Figure 4:**
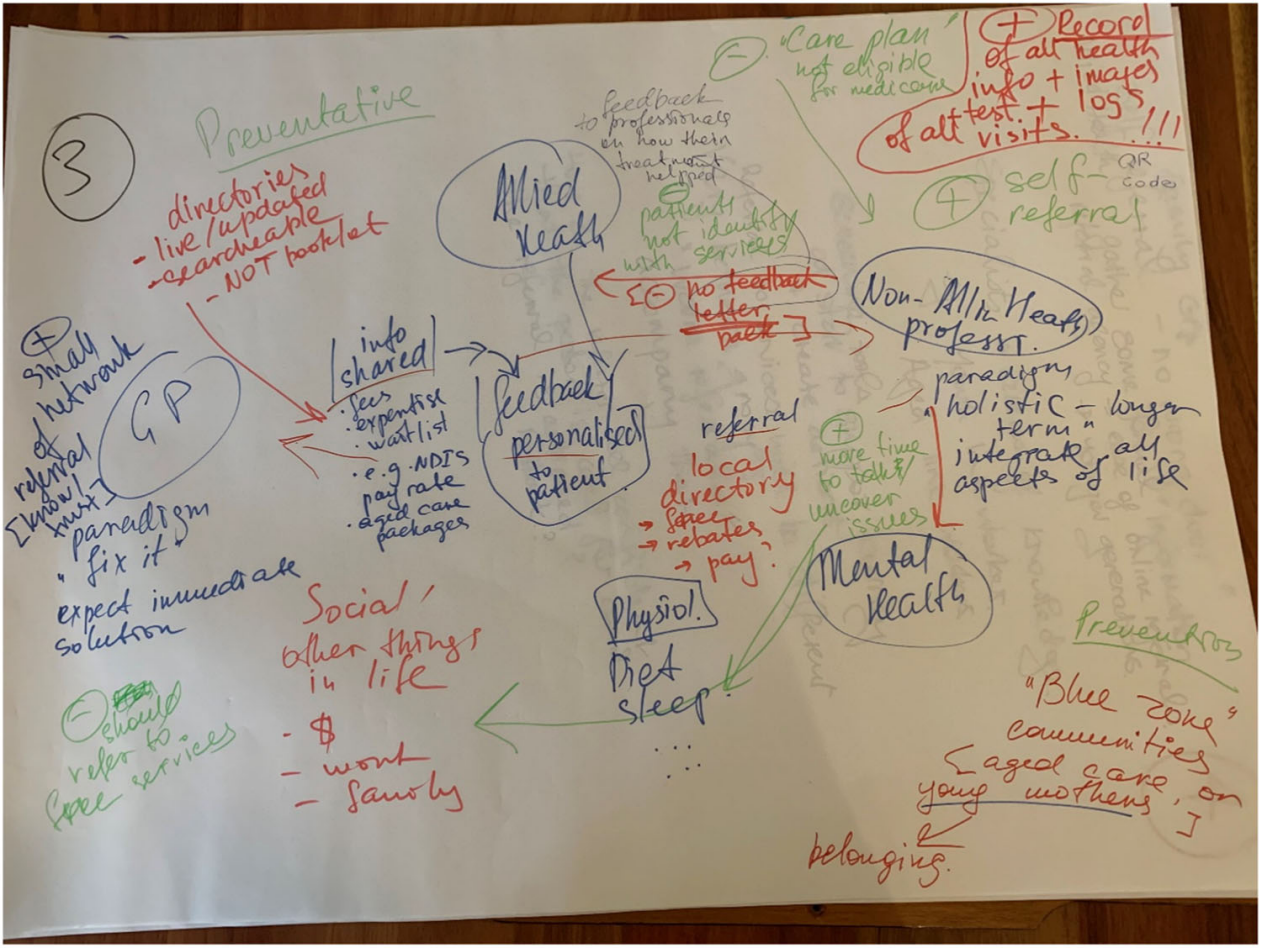
Example butcher paper ideas Service Provider Workshop 1.

Participants were then divided into groups of 3–5 people. Using butcher paper (805 × 565 mm sheets of blank paper), coloured pens, and sticky notes, they were invited to develop their own ideas for a social prescribing model of care (see Figure 4). Each group was facilitated by a member of the steering committee, who took notes during the discussion, helped the group to stay on task and ensured each group member had a voice. Each group was invited to present their ideas to the larger group for further discussion.

**Figure 5:**
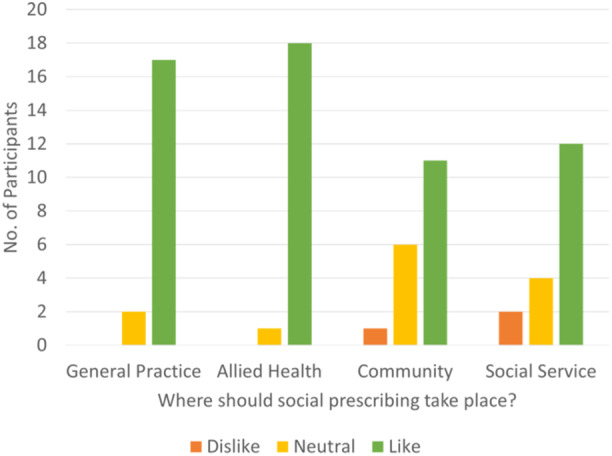
Example of workbook data results.

All the data were captured (via photos of the created idea ‘mud maps’ (visual representation of participants ideas)/butcher papers, completed booklets, and facilitators’ notes) and analysed for key themes and ideas. Quantitative workbook data was analysed descriptively (see example in Figure 5).

Qualitative data were summarised descriptively to represent what participants wanted the social prescribing model of care to look like. Four elements of the draft social prescribing model were identified:

1. *No wrong door*: in which the social prescribing programme is available to anyone with nonmedical needs with entry via general practice, allied health, community and self‐referral.
2. *Link worker is key*: where the link worker role was identified as fundamental to the programme to engage with the person over time and actively support them to connect with services and community. Participants suggested the need for multiple and diverse link workers to support the needs of particular population groups.
3. *Feedback loops*: where health and social care providers referring into the programme identified the need for information about whether and how the person they referred is being supported by the link worker.
4. *Supported by technology*: where the model of care is supported by social prescribing technology (e.g., an App), including an online care planning tool and maintained directory of social and community services.

**Figure 6:**
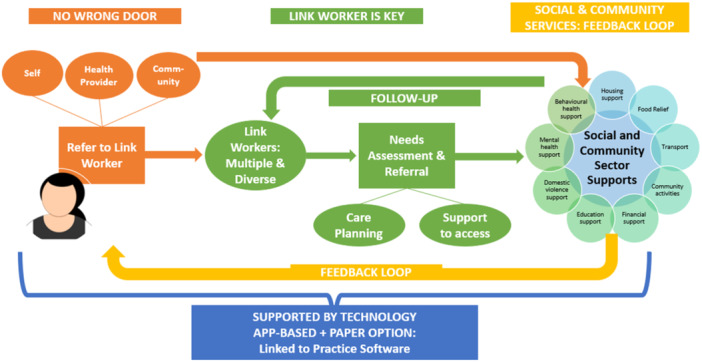
Draft model of care/client journey.

Results were brought together into a draft model of care/client journey (see Figure 6).

### 2.5 Service Provider Workshop 2: Validating the draft model of care and further refinement

A further service provider workshop was held to refine the model of care. The workshop was open to attendance by those participating in Workshop 1 in addition to those who expressed interest but were unable to attend the first workshop (*n* = 12, 75%, attended both workshops: one GP, seven allied health providers and four social service providers). The focus was on presenting the initial model to sense check that it correctly represented the views from the first workshop, to check if any critical elements were missing, and to workshop practical implementation of the proposed steps of the model. Sensitising for this workshop involved presenting a PowerPoint showing the results from the Workbooks, co‐design activity and discussions from Workshop 1, the draft model of care and the co‐design process.

**Figure 7:**
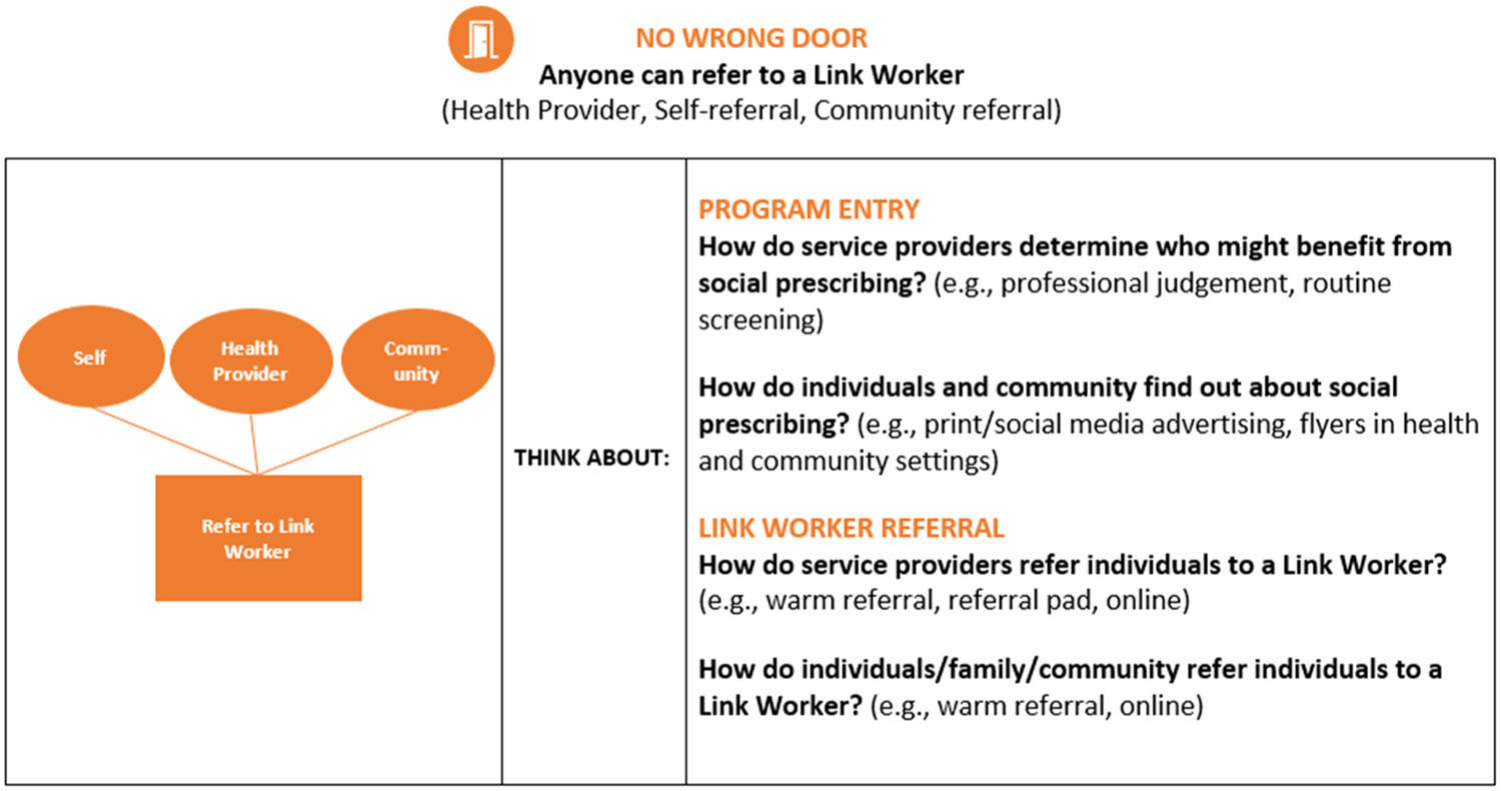
Example facilitator guide Service Provider Workshop 2.

Following the sensitising presentation, workshop participants were asked to individually provide written response to the following statements ‘Social prescribing would help me by …’, ‘Social prescribing would help my clients by …’ and ‘Social prescribing would help my community by …’. They were then asked to anonymously vote on the proposed draft model of care using a QR code linked to a question asking them to indicate whether they liked, disliked, or felt neutral about the draft model (94%, *n* = 15 liked the model with one participant voting ‘neutral’). They were then divided into groups of 3–5 people, each group focusing on one stage of the model. Groups were provided with examples of each stage in the model from other programmes (e.g., examples of needs analysis surveys, directories of services, care planning tools, social prescribing technology). Using butcher paper, coloured pens, and sticky notes, each group was asked to explore what could or should happen in each stage and present their ideas to the larger group for discussion. Groups were provided with a guide with questions about each stage (see example in Figure 7 and Supporting Information S1: Data), with facilitators from the steering committee taking notes on the discussions.

**Figure 8:**
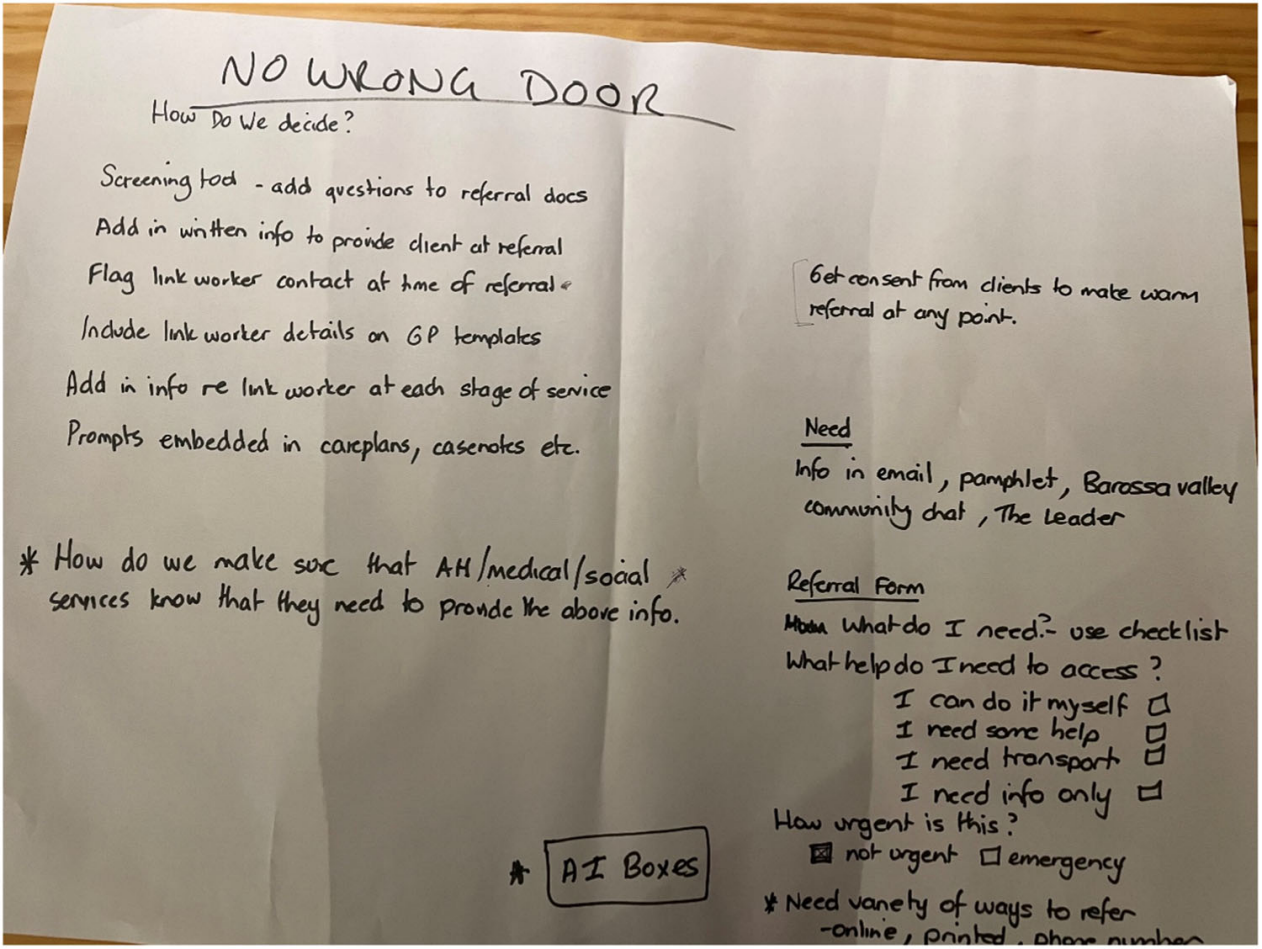
Example butcher paper ideas Service Provider Workshop 2.

Data in the form of butcher paper images (see example in Figure 8) and facilitator notes were analysed alongside data from community workshops to create the final social prescribing model of care (see below).

### 2.6 Community member Workshops 1 and 2: Feedback and refinement of the draft model of care

**Figure 9:**
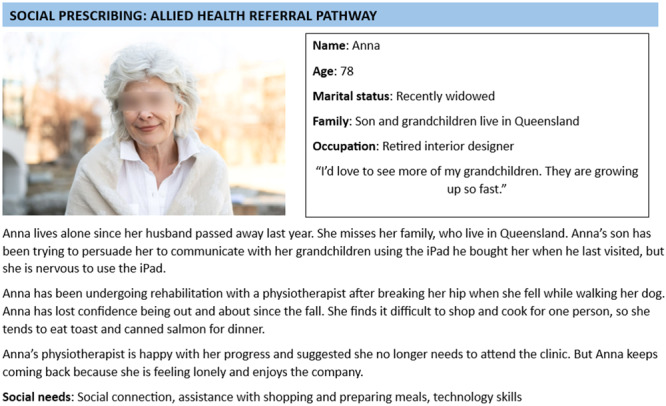
Example case study ( the image is a stock image from Microsoft Word, masked as per journal guidelines).

Case studies were developed of people in the region who have experienced nonmedical needs, based on examples provided in the service provider workshops with details changed to preserve anonymity (see example in Figure 9 and Supporting Information S1: Data). The draft model of care from service provider workshops was used to develop journey maps for community workshops. Five journey maps were developed, each based on a case study depicting typical circumstances and associated needs (see Supporting Information S1: Data).

Sensitising for community members involved a PowerPoint presentation describing the concept of social prescribing (including the video discussed above), presenting two of the case studies, the draft model of care, and explaining the co‐design process. Following the presentation of the case studies, participants were invited to discuss the case studies as well as their own experiences of social needs or those of others they knew or had heard about in groups of 3–5. The purpose of the discussion was to aid reflection on what social prescribing might mean for their community.

**Figure 10:**
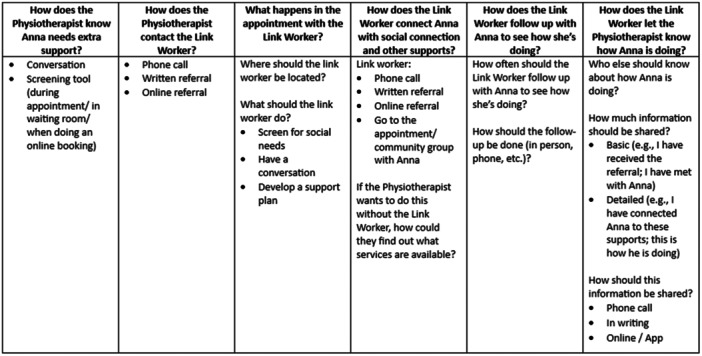
Journey map (with facilitator prompts).

Following the sensitising presentation and group discussion of the case studies, each group was provided with a case study, journey map (printed in A1 size), sticky notes and facilitator guide with ideas for each stage of the journey map (see Figure 10 for an example journey map with facilitator guide). Participants were guided through the task of filling in the social prescribing journey map for each case study by a facilitator from the steering committee, describing what they would like to happen at each stage of the journeys.

**Figure 11:**
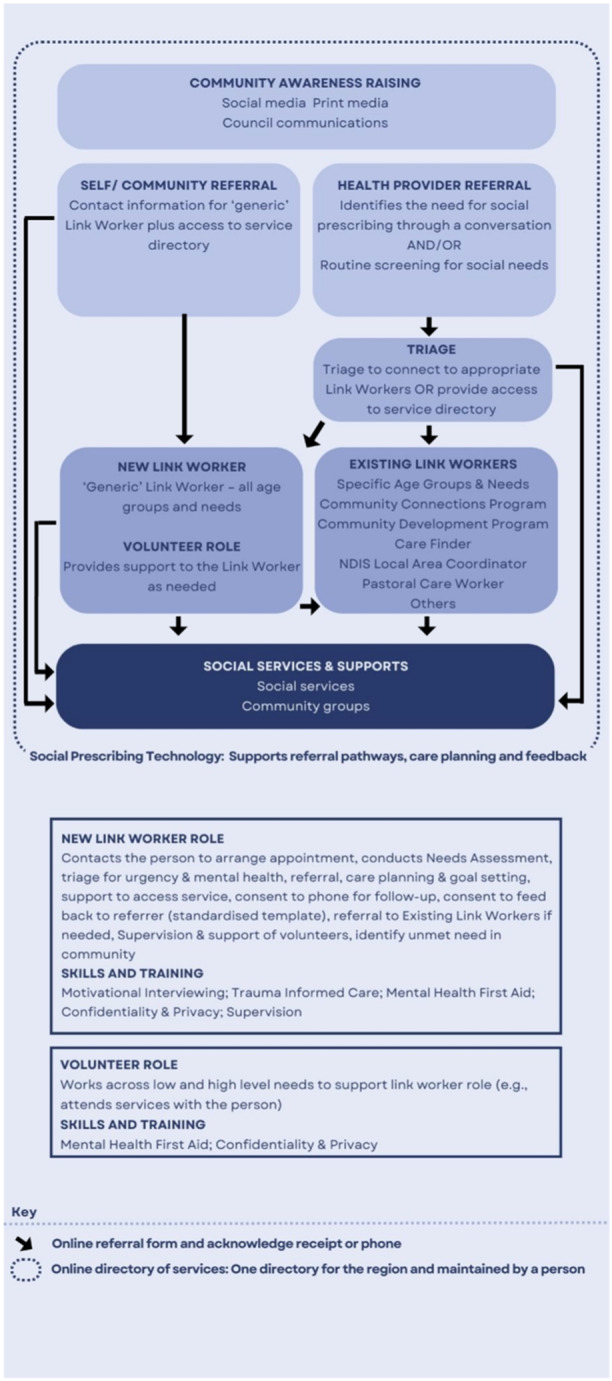
Co‐design social prescribing model of care.

Data in the form of completed journey maps and facilitator notes were analysed alongside data from service provider workshops to create the final social prescribing model of care and actions/activities at each stage in the model (see Figure 11). Following completion of workshops and data analysis, all participants were sent a summary of the outcomes and informed about the next steps in advancing social prescribing in the region.

#### 2.6.1 Building for change

As discussed by Trischeler et al.,^16^ ^,p.1612^ the outcome of co‐design is not expected to be a ‘market‐ready’ solution. Instead, it forms the basis for ‘an open dialogue between the researchers, partner organisations, and front‐line staff to assess the feasibility and realization of the ideas’. In addition to ongoing discussions during steering committee meetings, in our project building for change will involve presentation of the co‐design results to local councils and key players in the development and delivery of health and social care (Local Health Network, Primary Health Network, Department of Human Services, etc.), planned for early 2024. The steering committee has also started discussions around possible funding for implementation and evaluation of the co‐designed model.

## 3 DISCUSSION

This article describes the process of co‐designing social prescribing in a regional area of Australia. As social prescribing gains traction internationally, it is important that programmes are co‐designed to meet the needs of key stakeholders and the local context. There is increasing discussion in the literature of how social prescribing programmes have been co‐designed. For example, Santos‐Tapia et al.^22^ describe the process of co‐creating a nature‐based social prescription intervention in Spain. In a published protocol article, Ostojic et al.^23^ describe how they propose to co‐design a social prescribing programme for children with cerebral palsy and their families. However, to date there is little detailed information on a process of co‐designing social prescribing programmes that encompasses the broad range of approaches to social prescribing. By demonstrating the process and materials used in our project, we aim to open the ‘black box’ of co‐design for social prescribing and provide ideas and resources for others to adapt and utilise.

The project followed Trischeler et al.'s^16^ co‐design steps, successfully recruiting, sensitising, and engaging key stakeholders to co‐design a social prescribing model of care for the region. Workshops were lively and dynamic, with participants commenting on the feeling that their perceptions, experiences and suggestions were genuinely listened to. The very high repeat participation in workshops for service providers confirms that providers enjoyed the process and valued the outcomes. There was great interest in continuing to work together to implement the final model of care. The use of co‐design tools in the form of the Ideas Workbooks, case studies, and journey maps successfully fostered creativity and engagement across the range of stakeholders.

Participants identified the need for a holistic model of social prescribing involving link worker support (augmented by volunteers), ‘no wrong door’ entry to the programme and feedback loops to those referring people into the programme. Technology was identified as an important enabler of social prescribing, in addition to an updated and maintained directory of services. These elements have been included in other social prescribing programmes internationally.^4^

A novel element of the social prescribing model of care that emerged through co‐design was the inclusion of existing programmes that support people with their nonmedical needs and triaging people into the relevant programme. In the first service provider workshop, participants suggested the need for multiple link workers to support the needs of particular population groups (such as young people, older people, etc.). In the second workshop, participants noted that a range of supports are already available in the region that are focused on specific population groups or needs, such as the Community Connections Program (a generic programme for adults aged 18–64), Care Finder (available to vulnerable older people) and the National Disability Insurance Scheme Local Area Coordinator (available to people with disabilities and their families). Triaging people into existing programmes, in addition to a new link worker role to support those not eligible for these programmes, was identified as important to avoid service duplication and support sustainability. Participants further identified the need for community awareness raising of the concept of social prescribing to support uptake and engagement due to the novelty of the terminology and concept in Australia. Finally, while social prescribing is commonly delivered via general practice,^15^, ^24^ participants identified the need for multiple referral pathways including those beyond general practice and through community and self‐referral.

As Trischeler et al.^16^ identified in their research, co‐design does not necessarily follow a linear trajectory from resourcing the project through to building for change. Instead, they identified that the ‘front‐end’ (resourcing, planning, and recruiting) often requires multiple iterations to address recruitment challenges and reflect on the data and build for change. For our project, the iterative nature of the ‘front‐end’ reflects the way in which a social prescribing model of care was itself iteratively developed through key stakeholder engagement. For example, unlike previous research,^23^, ^24^, ^25^ there was no predetermined focus on a particular population group or specific nonmedical needs to be addressed through social prescribing. We began instead with a ‘blank slate’, presenting the components of social prescribing identified in our scoping review^4^ and allowing health and social service providers the freedom to design a draft model of care based on their experiences of the community and the needs they see in their practice. This provided the basis for resourcing, planning, and recruiting for subsequent workshops.

Unlike Trischeler et al.'s^16^ co‐design steps, where sensitisation and facilitation are presented as linear processes leading to reflection and building for change, in our project we responded to the process and outcomes of each workshop to determine the sensitising and facilitation of subsequent workshops in an iterative manner. The first service provider workshop drew on the needs analysis and scoping review for sensitisation, with facilitation following the planning and process stages identified in the scoping review.^4^ The second provider workshop involved presenting the outcomes of Workshop 1 and the use of examples from other social prescribing programmes related to each stage of the draft model of care to stimulate discussion. The first two service provider workshops then generated local case studies that were used in the community workshops to sensitise participants to the need for social prescribing. Facilitation for community workshops involved turning the case studies into journey maps to stimulate discussion.

Trischeler et al.^16^ ^,p.1614^ further note that the ‘back‐end’ (reflecting and building for change) is ‘a collaborative and iterative effort aimed at conceptualising viable solutions with key stakeholders’ rather than an end stage of idea evaluation. As a complex intervention that attempts to join up Australia's fragmented health and social care systems, building for change requires buy‐in from key stakeholders from both sectors. Our co‐design process served as a critical mechanism for raising awareness, interest and buy‐in to social prescribing by the key stakeholders. An important side effect of the workshops was that many stakeholders who have previously never heard of each other were able to find common goals and mechanisms for ongoing collaboration. The co‐design workshops also provided networking opportunities for key stakeholders, providing viable referral pathways that can be implemented in the interim.

This project highlights the importance of close collaboration with key stakeholders throughout the process, from conceptualisation through to realisation. The steering committee, with representatives across relevant key stakeholder groups, was central to the process of co‐design in this project. Together with the researchers, the steering committee developed the theory of change and project logic, participated in the ‘front‐end’ of co‐design to support resourcing and recruiting, co‐facilitated the workshops, and collaborated in (and continue to collaborate in) reflecting and building for change. Key stakeholder mapping throughout the iterative process across workshops further ensured involvement of relevant stakeholders.

One potentially contentious aspect of the process was the decision to conduct separate workshops with service providers and community members. Co‐design workshops are often conducted with mixed groups of service providers and community/consumers.^26^, ^27^ While commentators raise the potential for power differentials (e.g., between health providers and health consumers) to affect the ability of all participants to have a voice in co‐design, careful facilitation can address this issue.^27^ Other reasons to conduct separate workshop are ‘when considering healthcare at transitions and the patient pathways is complex’.^28^ ^,p.2130^ As discussed, the decision to conduct separate workshops was due to social prescribing being an unfamiliar concept to community members and the need to ensure the co‐designed model of care would fit with existing practices across health and social care services. Beginning with community members, or holding combined workshops, might have led to a different outcome and warrants further research.

## 4 CHALLENGES AND LIMITATIONS

The co‐design approach required strong engagement with stakeholders and community buy‐in and was relatively resource heavy. While service providers were keen to take part, it was difficult to find times and locations that allowed all interested providers to attend. We were not successful in recruiting younger people in the community workshops. While this group was not a specific focus, it is a limitation of the project. A further limitation is that due to participants self‐selecting to participate in workshops, community workshops participants are unlikely to be a representative sample of the community. Research specifically seeking the voices and experiences of marginalised communities is also needed in social prescribing co‐design.

While the iterative nature of the approach is a strength, in that it allowed for responsiveness to participant input in successive workshops, this limits the duplicability of the approach. Instead, the process followed, tools used and decisions made are presented as a guide for adaptation and judgement when reproduced in different contexts.

## 5 LESSONS LEARNED AND NEXT STEPS

This study provided important lessons for the researchers and steering committee, as follows:

1. The importance of strong key stakeholder engagement on the Steering Committee.
2. The need to develop relationships with GPs to engage them in social prescribing and co‐design.
3. The need to set clear time limits on co‐design activities to keep the workshops on track and allow sufficient time for each activity.

In terms of next steps, research is currently underway to co‐design and test the proposed triage process to determine appropriate link worker referral and to develop a campaign for awareness raising about the concept of social prescribing. Furthermore, given ongoing discussion in the literature of social prescribing outcome measurement,^29^ further co‐design is needed on how outcomes could be measured for the model.

## 6 CONCLUSION

Social prescribing offers the potential of better supporting people's social needs and improving health and wellbeing. There is growing interest in the design, implementation, and evaluation of social prescribing in Australia. Workshops with service providers and community members, adapting Trischeler et al.'s^16^ seven step process, were a successful platform for key stakeholders to co‐design a social prescribing model of care for the community. The processes, materials, and co‐design experience presented in this article provide guidance for social prescribing co‐design that can inform future research.

## AUTHOR CONTRIBUTIONS

**Candice Oster**: Conceptualisation; investigation; funding acquisition; writing— original draft; methodology; formal analysis. **Ashleigh Powell**: Investigation; writing—review and editing; formal analysis. **Claire Hutchinson**: Investigation; writing— review and editing; formal analysis. **Debra Anderson**: Conceptualisation; investigation; funding acquisition; writing—review and editing. **Bill Gransbury**: Conceptualisation; investigation; writing—review and editing. **Martin Walton**: Investigation; writing—review and editing. **Jenny O'Brien**: Investigation; writing— review and editing. **Susan Raven**: Investigation; writing—review and editing. **Svetlana Bogomolova**: Investigation; methodology; writing—review and editing; formal analysis.

## CONFLICT OF INTEREST STATEMENT

The authors declare no conflict of interest.

## ETHICS STATEMENT

The study was approved by the Flinders University Human Research Ethics Committee (Project number 4868). Potential participants were provided an information sheet explaining the project and their involvement. Those who agreed to participate were asked to sign a consent form.

## Supporting information

Supporting information.

## Data Availability

The anonymised data that support the findings of this study are available on reasonable request from the corresponding author. The data are not publicly available due to privacy and ethical restrictions.
