# Supplementary material for: The process of co‐designing a model of social prescribing: An Australian case study
Source: Health Expect. 2024 May 24;27(3):e14087. doi: 10.1111/hex.14087 (PMC11116944; doi:10.1111/hex.14087)
Supplement: Supplementary file 1 — Supporting information. [file HEX-27-e14087-s001.docx]

**The process of co-designing a model of social prescribing: An Australian case study**

**Supplementary file**

**Contents**

- Project logic and Theory of Change
- Health and Social Service Provider Ideas Workbook: Workshop 1
- Health and Social Service Provider Ideas Workbook: Workshop 2
- Health and Social Service Provider Small Group Activity: Workshop 2
- Community Workshops: Case Studies and Journey Maps with Facilitator Guides

**SOCIAL PRESCRIBING CO-DESIGN**

**PROJECT LOGIC & THEORY OF CHANGE**

| **Objective:** To co-design a model of social prescribing with health professionals, service providers and community members in the region. | | | | | |
| --- | --- | --- | --- | --- | --- |
| **Needs statement**  Residents are experiencing unprecedented financial stress, homelessness, food insecurity, and loneliness/social isolation. Social prescribing provides non-clinical referral pathways for residents to access support for social needs. It is important for social prescribing to be co-designed by key stakeholders, including health professionals, service providers and community members. | | | | | |
| 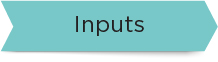 | 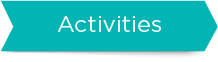 | 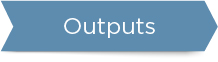 | 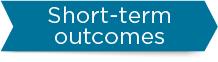 | 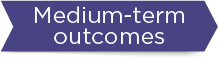 | 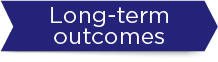 |
| Funding from SA Healthy Towns Project.  Project committee:   - Led by Local Government - Cross-sectoral representation - University   Strong networks across the region.  Knowledge & expertise in co-design.  Knowledge of components and models of social prescribing.  Qualitative and quantitative data from needs assessment. | Project committee workshop  Co-design service workshop 1: health and social service providers (draft model).  Co-design workshop 2: health and social service providers (model refinement).  2 x Co-design workshops with community (model feedback and input).  Information session with service providers not engaged in the co-design process. | Co-designed social prescribing model.  Initial development of a directory of social and recreational supports that can be utilised as part of the model. | Health Professionals and Service Providers:   - Understanding the effects of social determinants on health and wellbeing. - Knowledge of components and models of social prescribing. - Having a voice in program design.   Community:   - Understanding the effects of social determinants on health and wellbeing. - Awareness of social prescribing. - Having a voice in program design. | Health Professionals and Service Providers:   - Strengthened cross-sectoral collaboration to address social determinants of health. - Increased knowledge of available community and social assets. - Commitment from service providers to support the model of social prescribing in roles that are relevant to their organisation. | Implementation and evaluation of the social prescribing model of care.  Community:  Improved health and wellbeing as a result of “filling social prescriptions”. |
| **Theory of change statement**  Australians are facing unprecedented financial stress, homelessness, food insecurity, and loneliness/social isolation. This crisis is exacerbated by the combined effects of the COVID-19 pandemic and the current cost of living, disproportionately affecting the most vulnerable Australians^1^. Social needs are best addressed by social and community support services. However, 6-36% of General Practice consultations are for social needs^2^ while social and community services experience difficulties connecting those at greatest disadvantage to their services^3^. There is a clear need for referral pathways between health services and the social and community service sectors to better support people experiencing social needs. This can be done through social prescribing, a systematic approach to screening people for social needs and referring them to social and community services^4^. Social prescribing is in its infancy in Australia relative to the UK and USA but is gaining momentum in practice and policy^5^. With the evidence-base for social prescribing coming from the UK and USA, co-design with key stakeholders is needed to ensure adequate fit with Australia’s health and social care systems and to ensure models of social prescribing meet the needs of Australian communities^6^.  This project will co-design a model of social prescribing with key stakeholders in the region. Co-design workshops will be conducted, two with health professionals and social service providers and two with community members. Assumptions of the project are that participation in co-design workshops will improve participants’ understanding of the effects of social determinants on health and wellbeing and their knowledge and awareness of social prescribing and provide them with a voice in co-design. It also assumes that participation in workshops will strengthen cross-sectoral collaboration to address social determinants of health and that service providers will understand and commit to their role in the social prescribing model. The outcome of the project will be a co-designed model of social prescribing for implementation and evaluation in a future project, followed by a long-term outcome of improved health and wellbeing for community members as a result of “filling social prescriptions”.  References:  1. Green H, Fernandez R, MacPhail C (2022). Well-being and social determinants of health among Australian adults: A national cross-sectional study. Health and Social Care in the Community, 30:e4354.  2. Royal Australian College of General Practitioners (RACGP) (2022). General Practice Health of the Nation 2022. RACGP, Australia.  3. Bogomolova S, Eyers-White D, Tan PJ, Jardine B, Mackenzie C, and Goodwin-Smith I (2022). "… it gave me the oomph to go and do it … or I'd still be sitting in the house 24/7.” Community Connections Program. Evaluation Report. Centre for Social Impact, Flinders University. Adelaide.  4. Morse DF, et al. (2022). Global developments in social prescribing. BMJ Global Health, 7:e008524.  5. Australian Government (2021). National Preventive Health Strategy 2021-2030. Australian Government, Canberra.  6. Oster C, et al. (2023). Models of social prescribing to address non-medical needs in adults: A scoping review. BMC Health Services Research, 23:642. | | | | | |

**SOCIAL PRESCRIBING CO-DESIGN**

**SERVICE PROVIDER WORKSHOP 1**

**INDIVIDUAL TASKS**

**Note**: Images are stock images from Microsoft Word. Images of people are masked as per journal guidelines.

Social Prescribing Co-Design

BOOKLET OF IDEAS

***Instructions: please review each page by yourself, answering any questions and noting your thoughts and comments on the margins.***

***(Print in colour)***

**Instructions: On the next few pages, please indicate your liking or disliking of each component of social prescribing shown in pictures (by circling the ‘faces’).**

**Feel free to add any other comments on the margins.**

**Start thinking which components you would like to see in a social prescribing programme.**

**SOCIAL PRESCRIBING PATHWAYS**


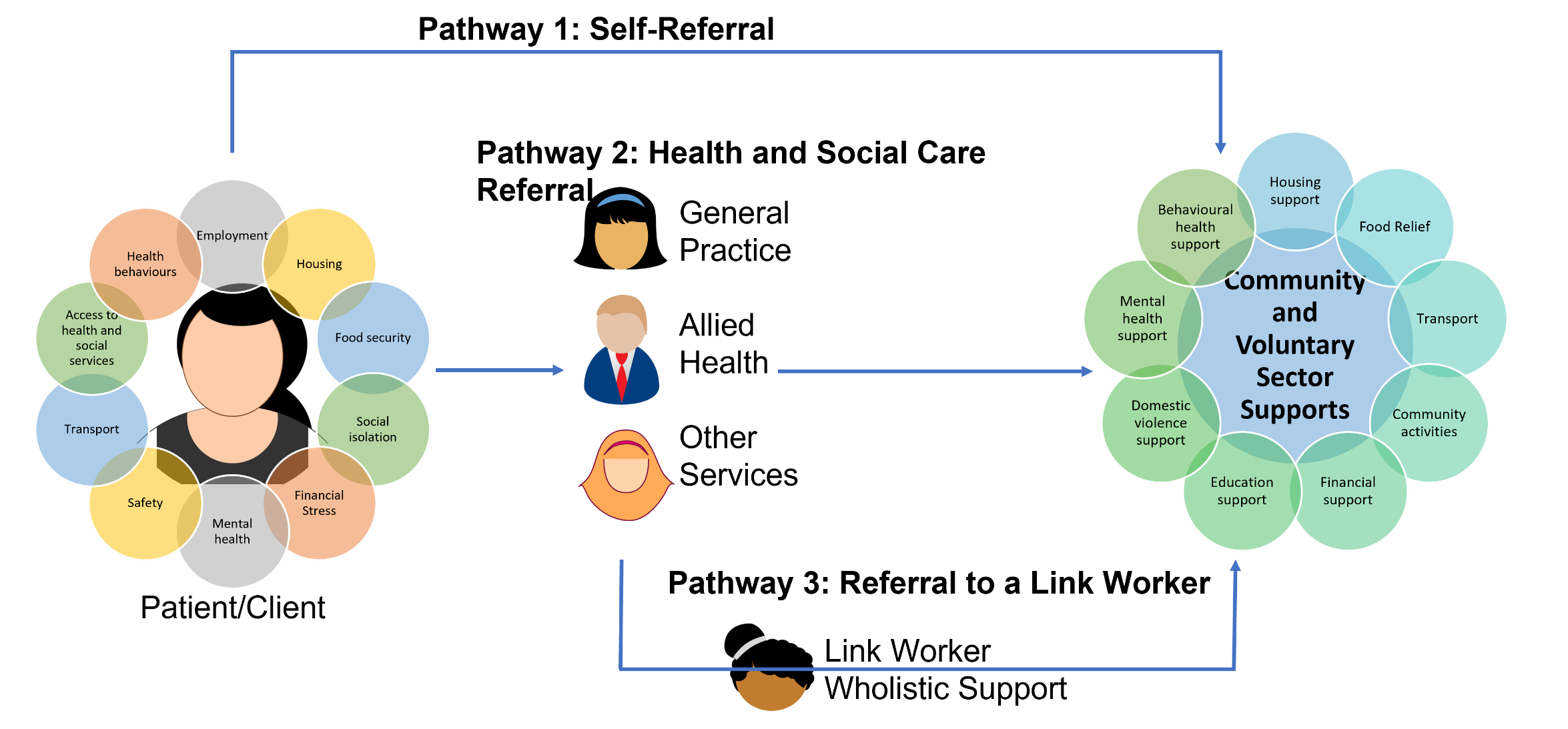


**WHICH SOCIAL PRESCRIBING PATHWAY?**

**(can be one, two, or all three)**

| 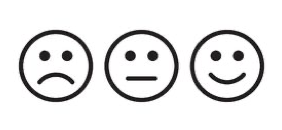 | 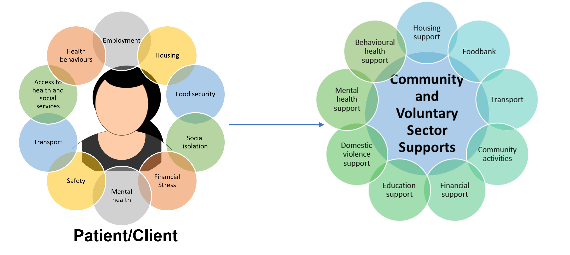  **Pathway 1: Self- Referral** | 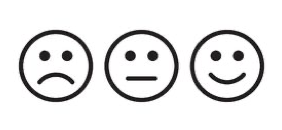 | 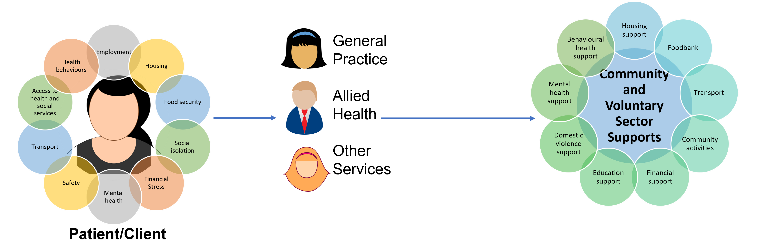  **Pathway 2: Health and social care referral** |
| --- | --- | --- | --- |
| 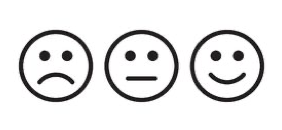 | 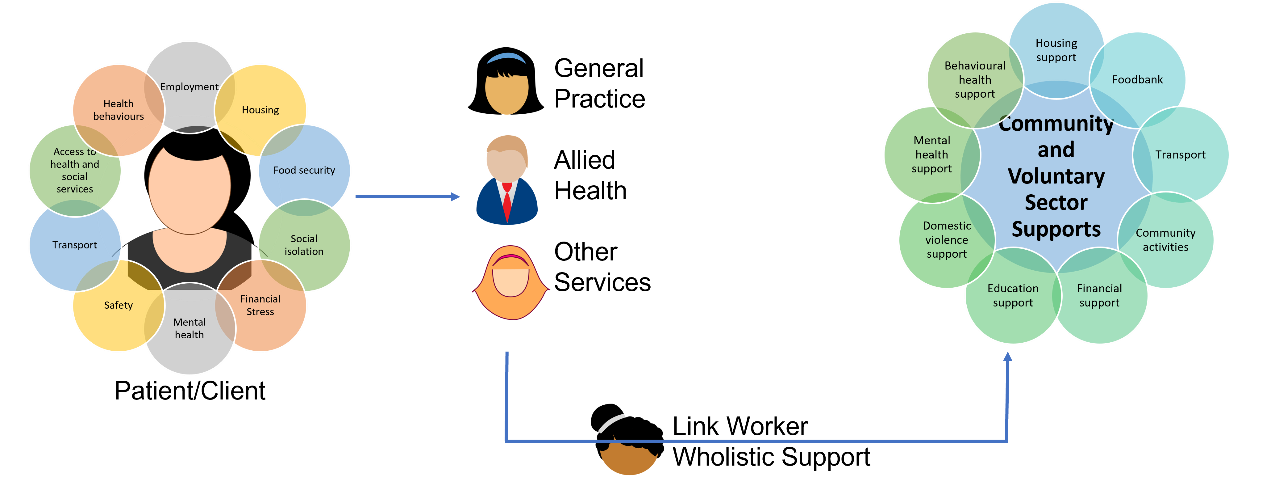  **Pathway 3: Health and social care referral to a Link Worker** | | |

1. Planning stages and decision-making

[Note: Identifying available services and supports will be discussed towards the end of the workshop]

**
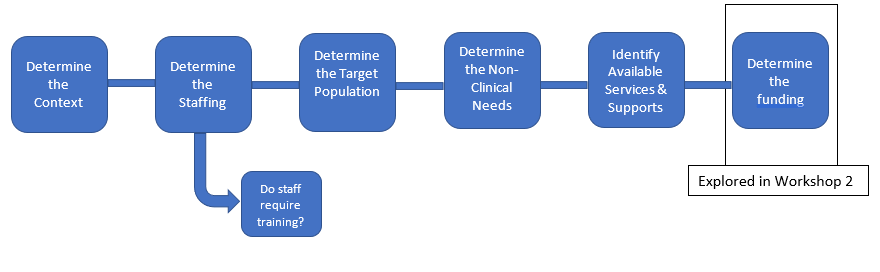
**

**WHERE DO WE WANT SOCIAL PRESCRIBING TO HAPPEN?**

| 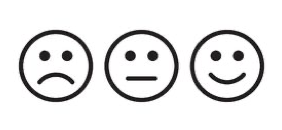 | *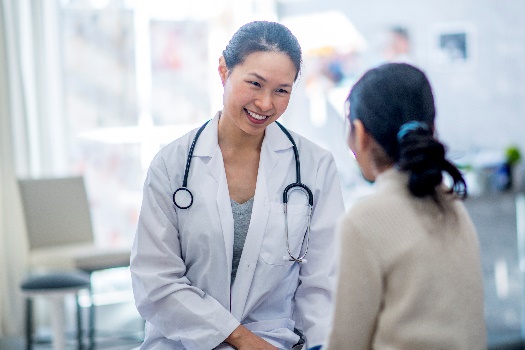*  **In General Practice** | 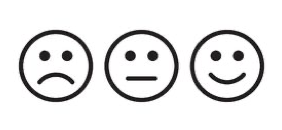 | 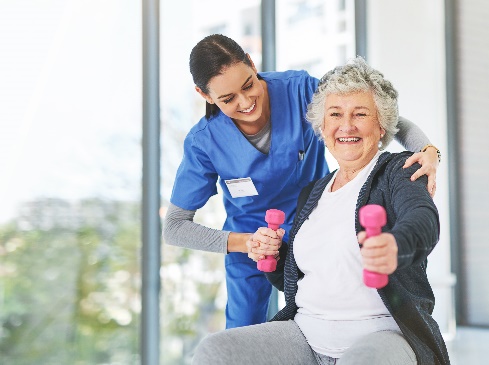  **In Allied Health Practices** |
| --- | --- | --- | --- |
| 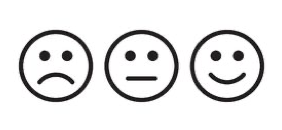 | *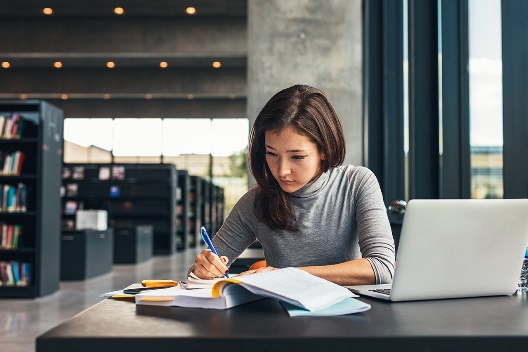*  **In Community Settings**  (E.g., Library, online) | 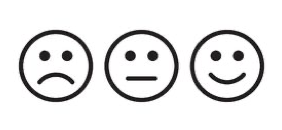 | *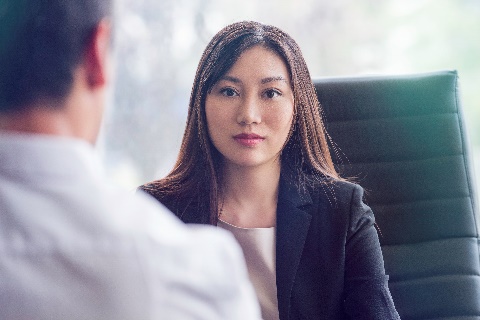*  **In Social Service Settings**  (E.g., Centrelink) |

**STAFFING THE SOCIAL PRESCRIBING MODEL?**

**(What staff are needed)**

| 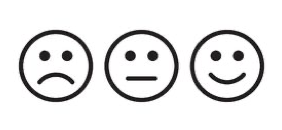 | **Existing staff**  (GP or allied health during routine consultation) | 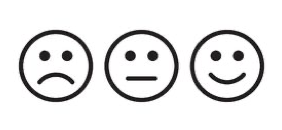 | **Nominated internal Link Worker**  (E.g., practice nurse) |
| --- | --- | --- | --- |
| 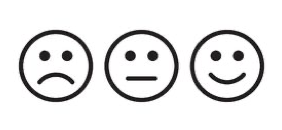 | **New Link Worker role**  (E.g., funded position/volunteer) | 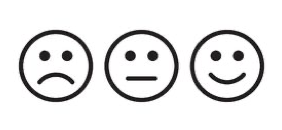 | **Existing Link worker role**  (Community Connections Program link workers) |

**STAFF TRAINING?**

| 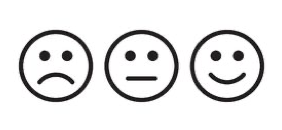 | **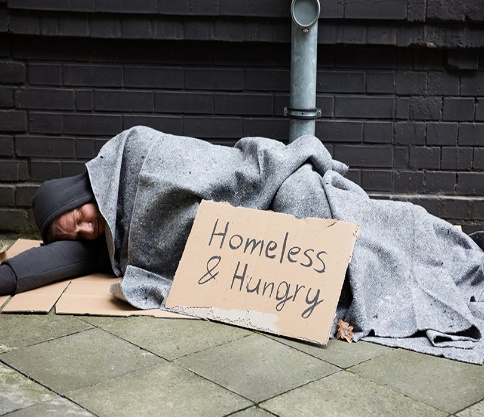**  **Training in the effects social determinants of health** | 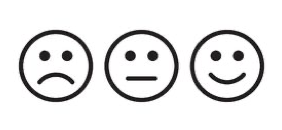 | **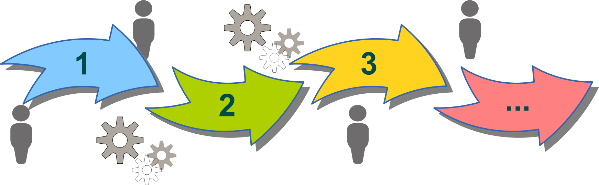**  **Training of staff in the model of social prescribing**  (Processes and procedures) |
| --- | --- | --- | --- |
| 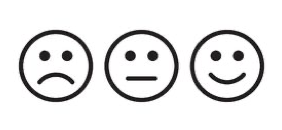 | **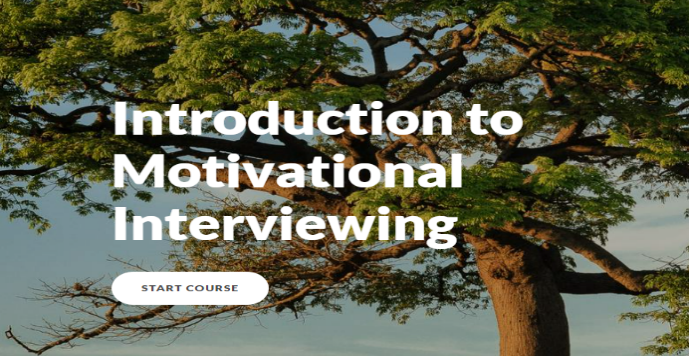**  **Training of Link Workers**  (E.g., Motivational Interviewing; goal setting; mental health first aid) | | |

**TARGET POPULATION**

**(Who will take part in the programme?)**

| 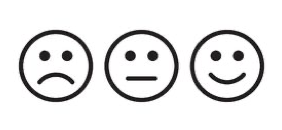 | 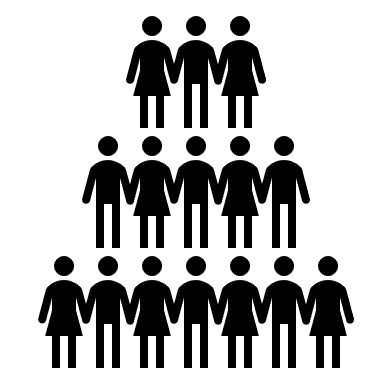  **General Population** | 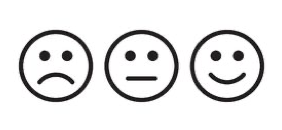 | 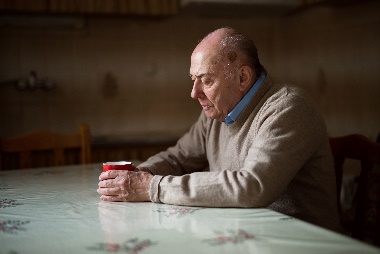  **Specific At-Risk Groups**  (E.g., People with long-term conditions; older people; people with mental health issues; etc.) |
| --- | --- | --- | --- |

**NON-CLINICAL NEEDS TO BE ADDRESSED?**

| 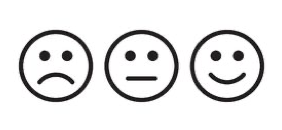 | **Social Determinants of Health** | 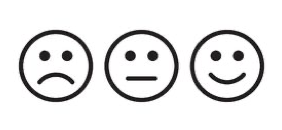 | *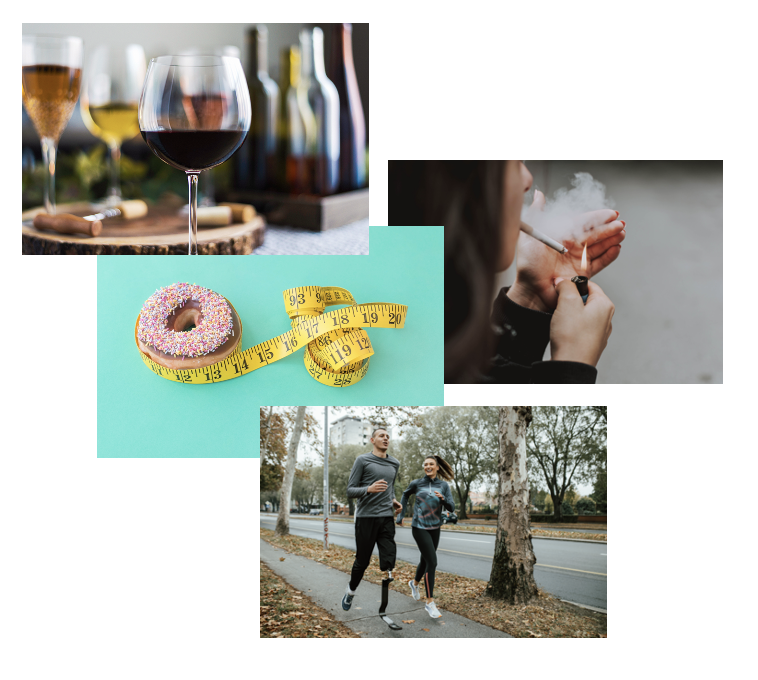*  **Health Behaviours**  (E.g., smoking, exercise, diet) |
| --- | --- | --- | --- |

2. Process stages and decision-making


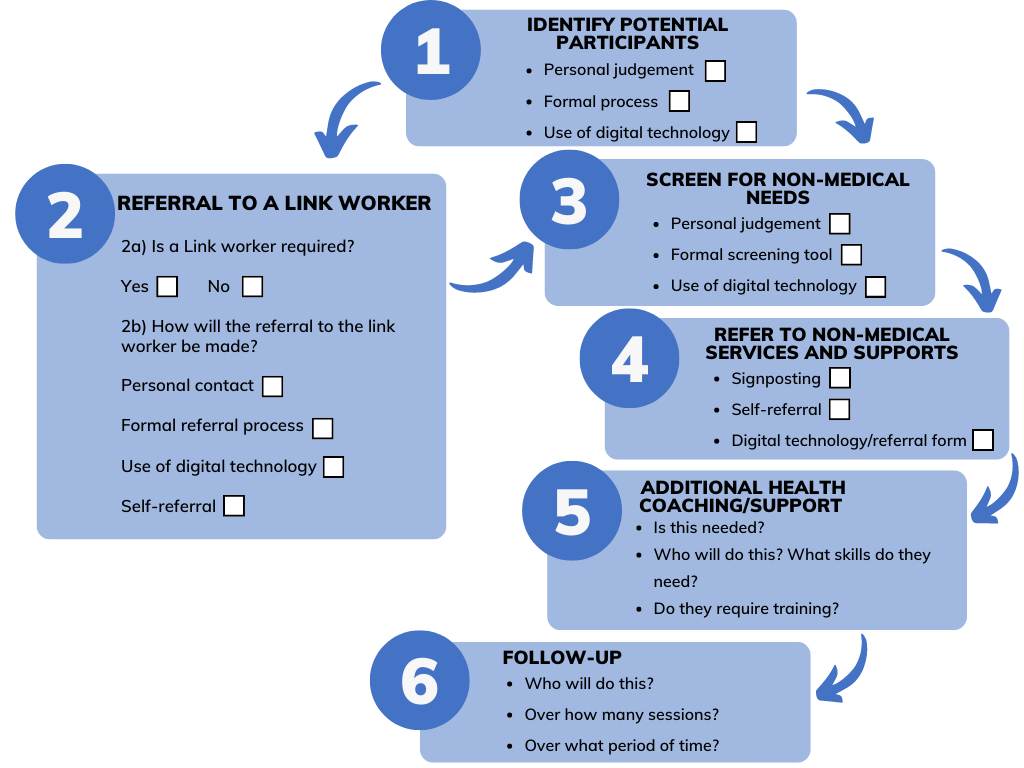


**HOW WILL PEOPLE BE IDENTIFIED FOR PARTICIPATION IN THE SOCIAL PRESCRIBING PROGRAM?**

| 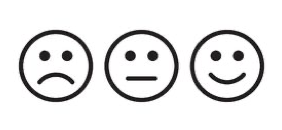 | 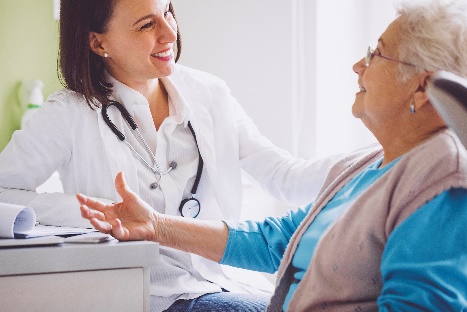  **Professional judgement**  (E.g., during a medical/allied health consultation) | 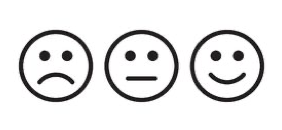 | 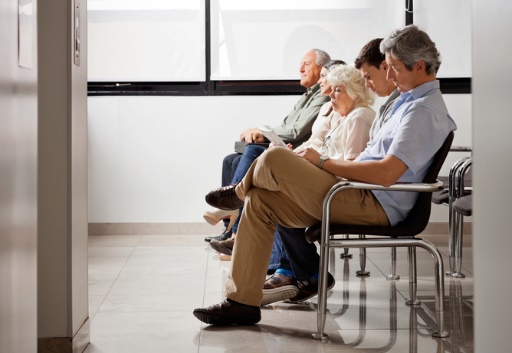 **Routine screening**  (E.g., in waiting room) |
| --- | --- | --- | --- |

**HOW WILL THE PERSON’S NON-CLINICAL NEEDS BE IDENFIFIED FOR REFERRAL?**

| 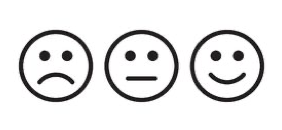 | 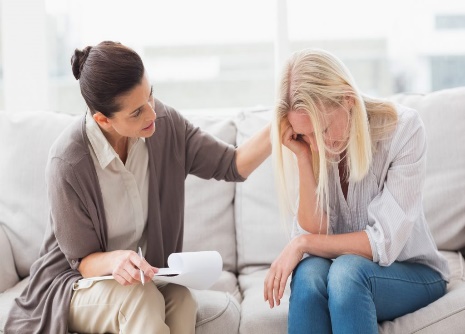 [This Photo](https://www.geoffreydromard.com/the-types-of-psychologists/) by Unknown Author is licensed under [CC BY](https://creativecommons.org/licenses/by/3.0/)  **Professional judgement**  (E.g., identify specific needs during consultation) | 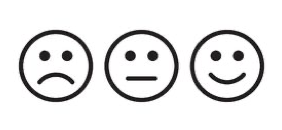 | *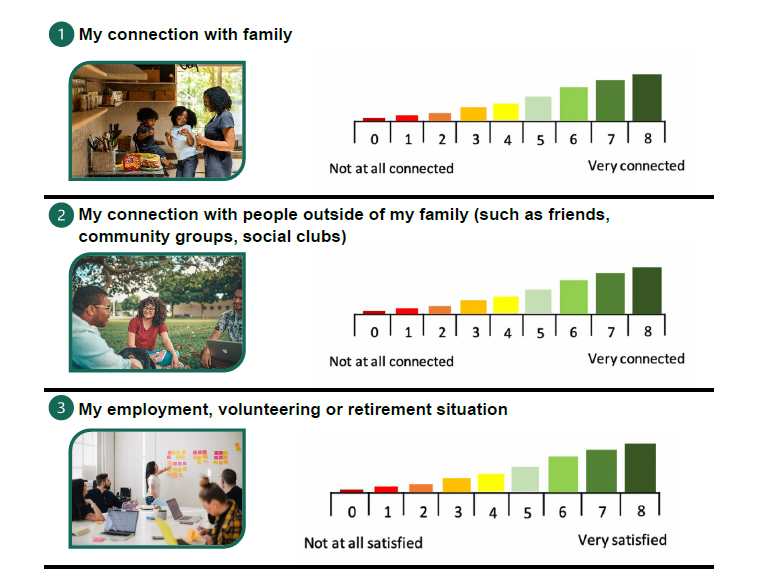*  **Use of a screening questionnaire** |
| --- | --- | --- | --- |
| 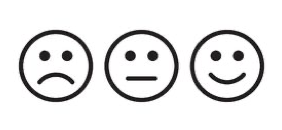 | 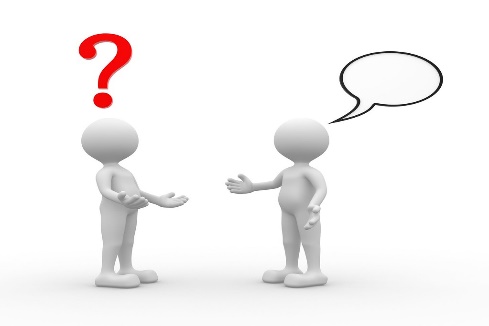 [This Photo](https://www.flickr.com/photos/60525373@N08/48227463931/) by Unknown Author is licensed under [CC BY-NC](https://creativecommons.org/licenses/by-nc/3.0/)  **Self-identification** | | |

**IF THERE IS A LINK WORKER: REFERRAL PROCESS?**

| **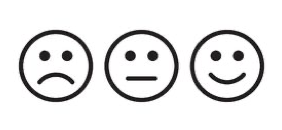** | 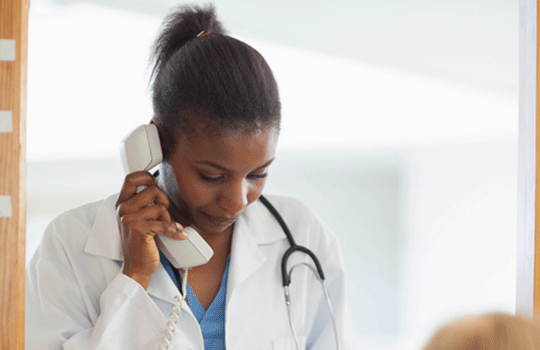 [This Photo](https://www.bmj.com/content/366/bmj.l4134.short?rss=1) by Unknown Author is licensed under [CC BY-NC](https://creativecommons.org/licenses/by-nc/3.0/)  **Warm Referral**  (Contacting the link worker for or with the client) | **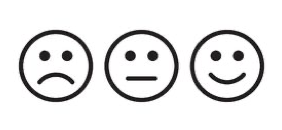** | **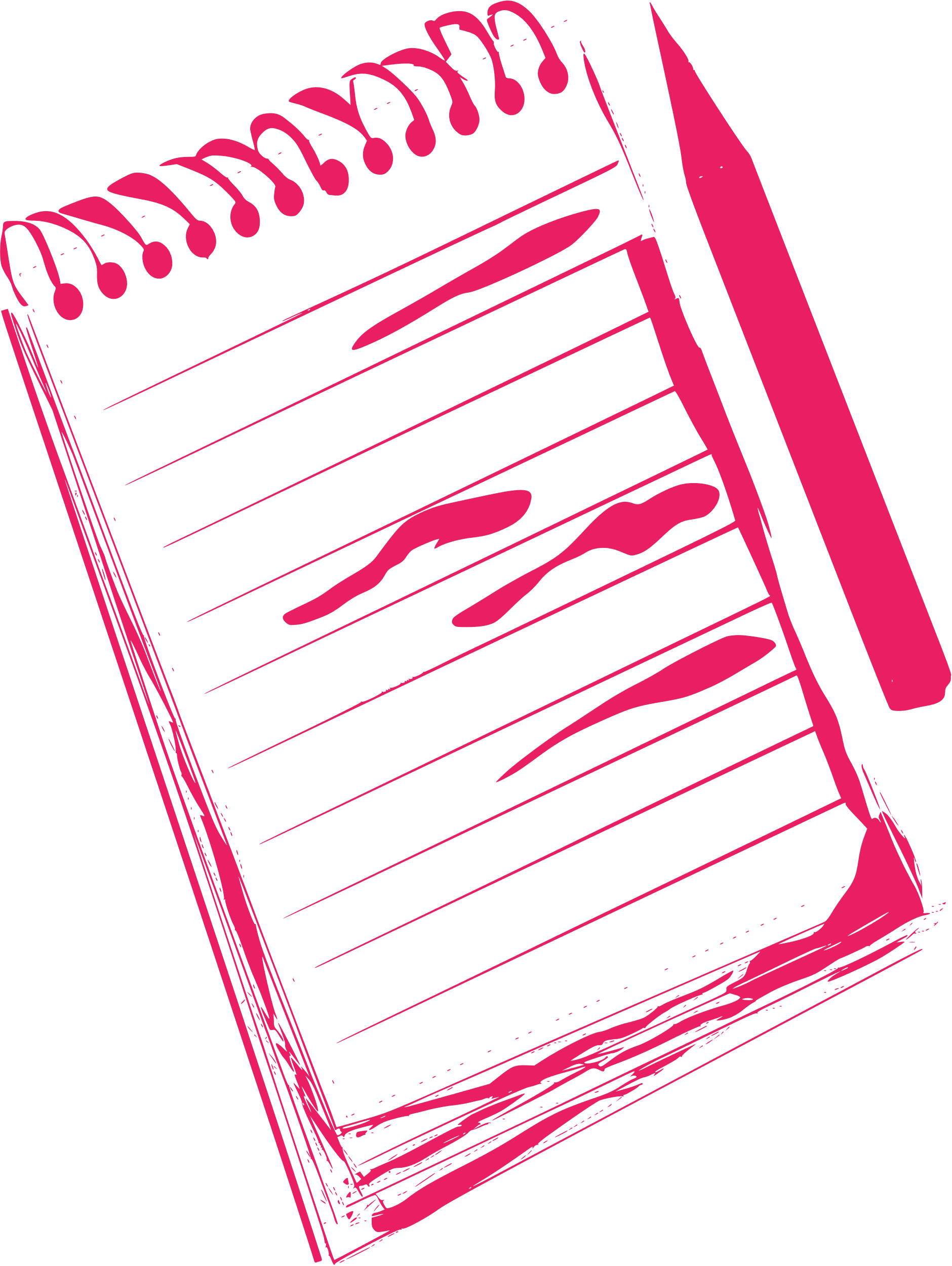**  **Referral pad**  (Client takes the referral to the link worker) |
| --- | --- | --- | --- |
| **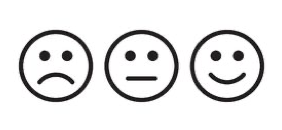** | **Self-referral**  (E.g., providing information about a link worker service) | | |

**REFERRAL PROCESS: REFERRAL TO SOCIAL/COMMUNITY SERVICES?**

| 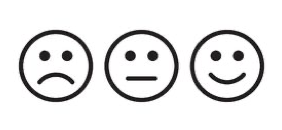 | 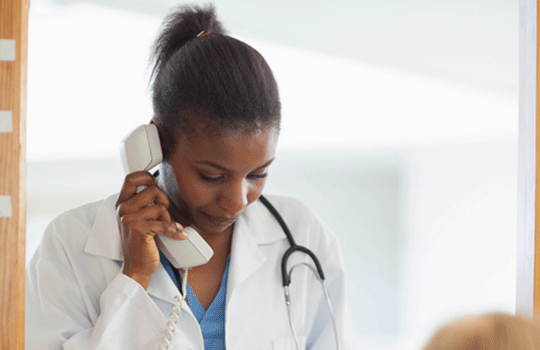 [This Photo](https://www.bmj.com/content/366/bmj.l4134.short?rss=1) by Unknown Author is licensed under [CC BY-NC](https://creativecommons.org/licenses/by-nc/3.0/)  **Warm Referral**  (Contacting a service for or with the client) | 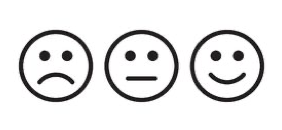 | 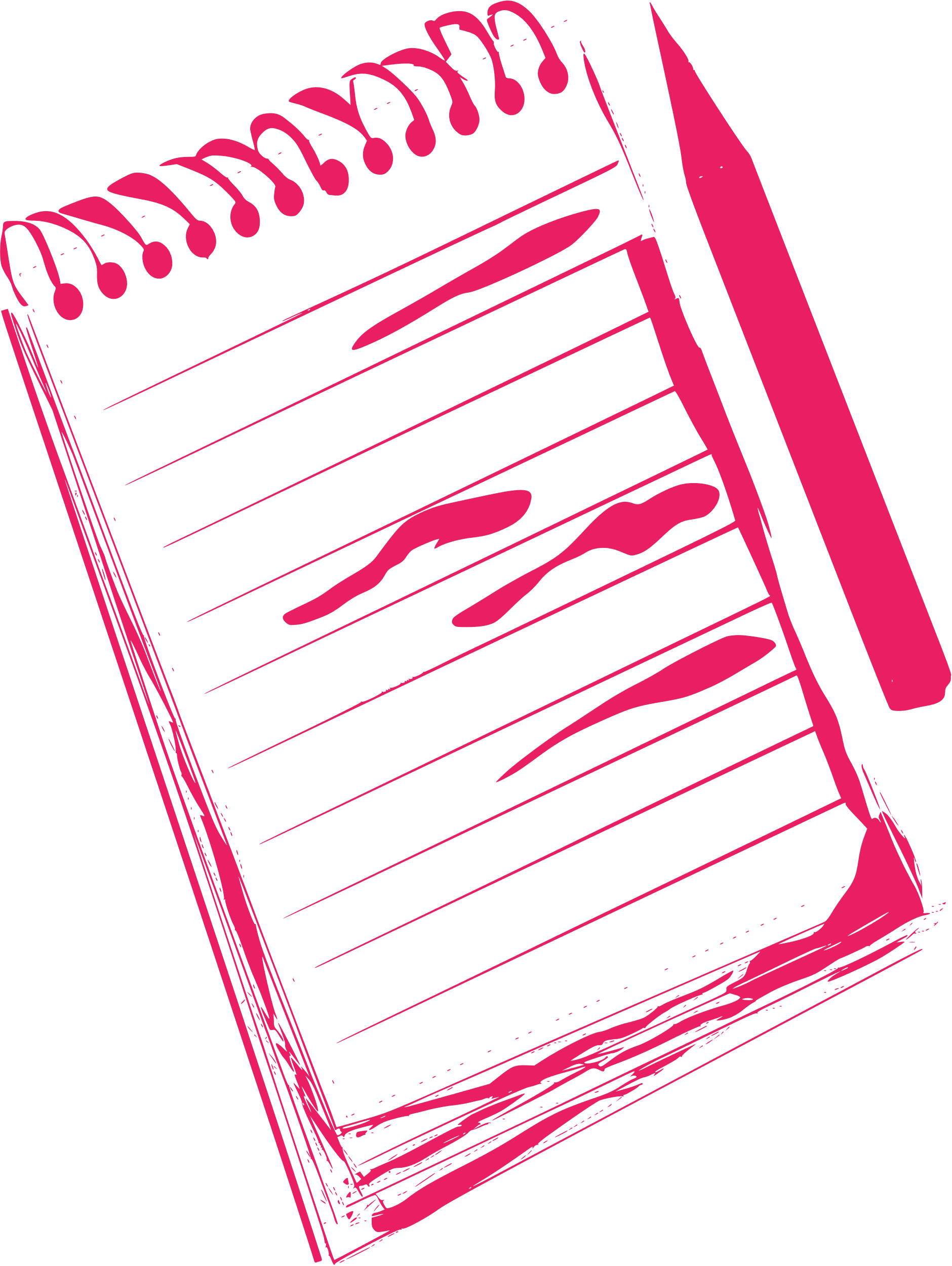  **Referral pad**  (Client takes the referral to the service) |
| --- | --- | --- | --- |
| 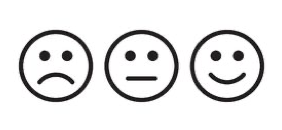 | 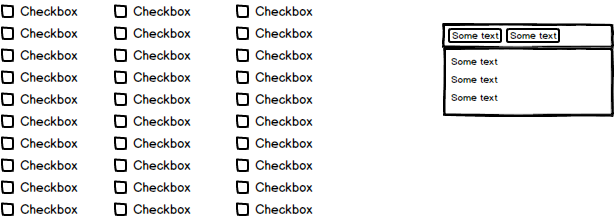 [This Photo](http://ux.stackexchange.com/questions/38264/list-of-checkboxes-or-multi-value-select-box) by Unknown Author is licensed under [CC BY-SA](https://creativecommons.org/licenses/by-sa/3.0/)  **Computer**  (E.g., tick-box and download a list of relevant services and contact information) | 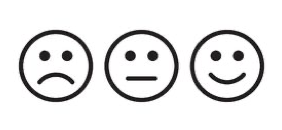 | *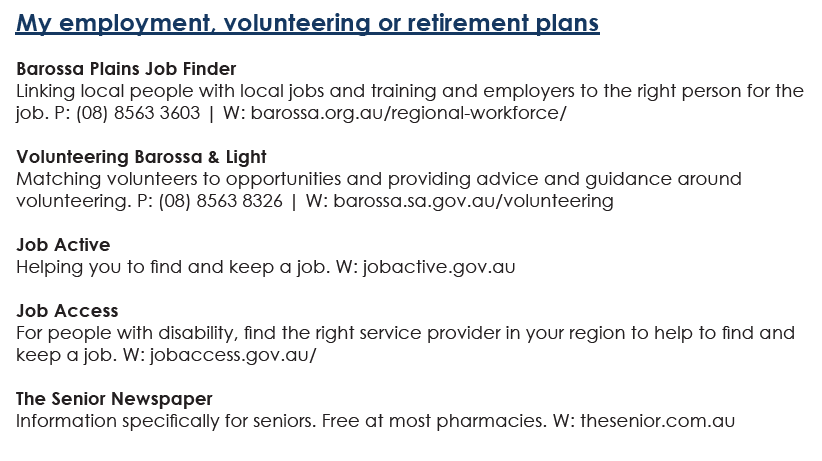*  **Self-referral**  (E.g., providing a booklet of services / online link to a service directory) |

**FOLLOW-UP?**

| 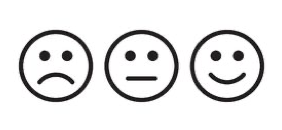 | **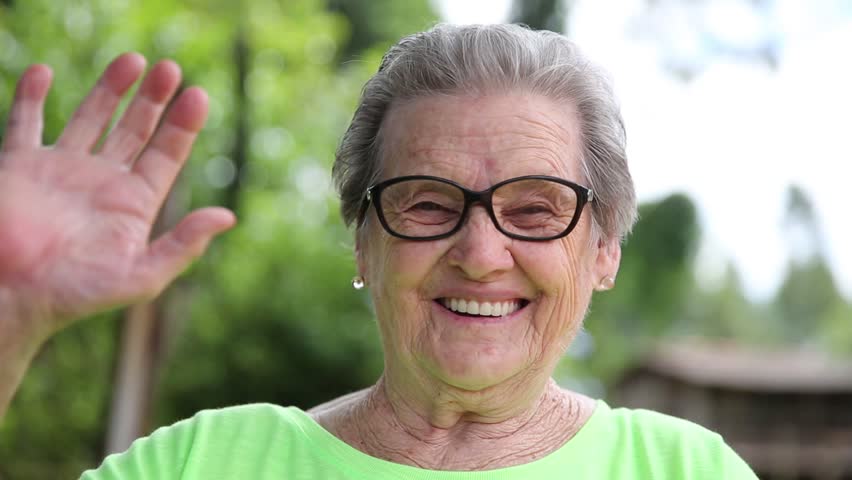**  **No follow-up** | 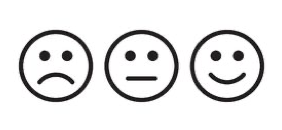 | 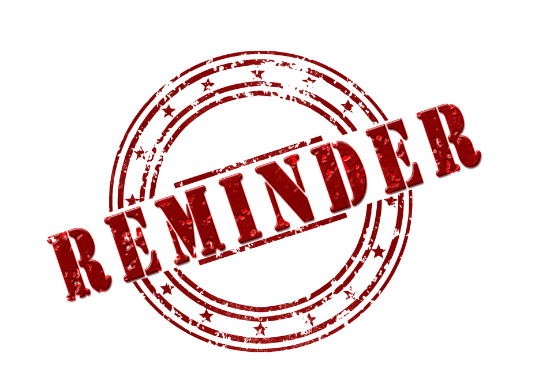  **Follow-up**  (E.g., reminder in the client information system) |
| --- | --- | --- | --- |

**TECHNOLOGY?**

| 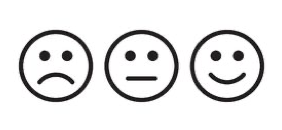 | *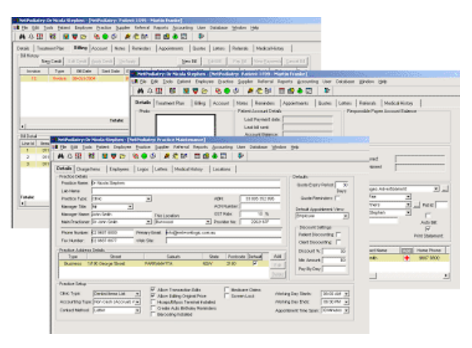*  **Including social prescribing in existing patient management software** | 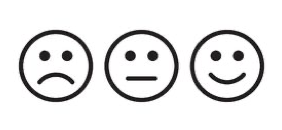 | *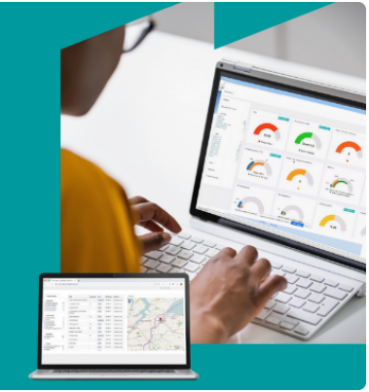*  **New software for social prescribing** |
| --- | --- | --- | --- |
| 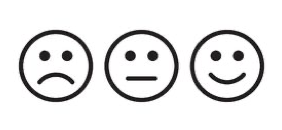 | **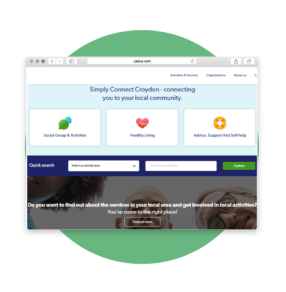**  **Public Facing Social Prescribing Referral Platform**  (E.g., Social needs screening survey  linked to a service directory; downloadable referral form) | | |

3. Developing and maintaining service database

| 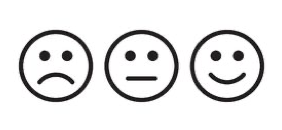 | 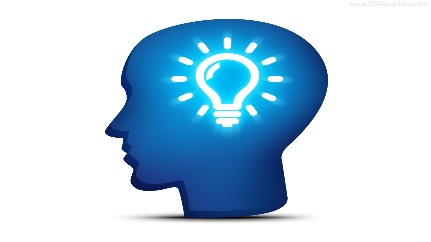 [This Photo](https://esheninger.blogspot.com/2017/05/rise-of-edupreneur.html) by Unknown Author is licensed under [CC BY](https://creativecommons.org/licenses/by/3.0/)  **Personal knowledge**  (E.g., of health/social care provider or link worker) | 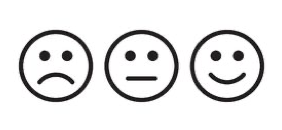 | 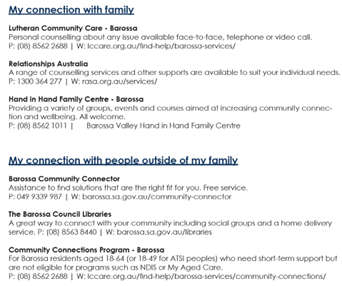  **Local service mapping and**  **Booklet of services** |
| --- | --- | --- | --- |
| 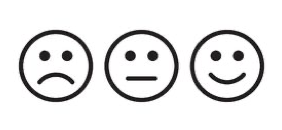 | 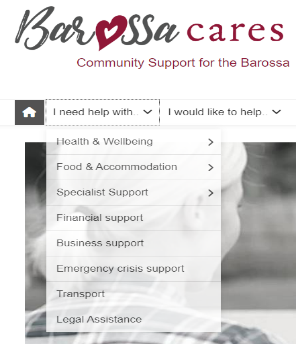  **Local service mapping and Online database** | 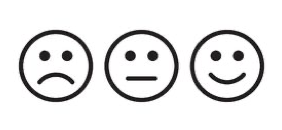 | 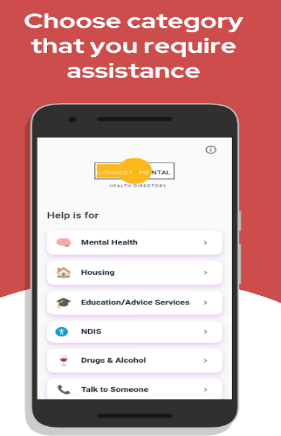  **Postcode Searchable Community Directory App/Website** |

SHORT QUESTIONS

(For statistical purposes only. Please write response or circle appropriate answer)

1. What is your profession?
2. About how long have you worked in your profession? A. 1 year or less B. 2-5 years C. >5 years
3. What is your gender? A. Male B. Female C. Non-binary D. Prefer not to answer
4. Which age group do you belong to?
5. Under 25yo B. 26-35yo C.36-45yo D.46-55yo E.56-65yo F.65+yo G. Prefer not to answer

Thank you for completing these questions. We will shortly return to the group discussion.

**SOCIAL PRESCRIBING CO-DESIGN**

**SERVICE PROVIDER WORKSHOP 2**

**INDIVIDUAL TASKS**

**SOCIAL PRESCRIBING CO-DESIGN**

**SERVICE PROVIDER WORKSHOP 2**

**INDIVIDUAL TASKS**

TASK 1: FINISH THE SENTENCES

*Instructions: Complete the unfinished sentences below with whatever ideas first come to mind.*

| **START** | **FINISH THE SENTENCE (WRITE DOWN)** |
| --- | --- |
| Social prescribing would help me by … |  |
| Social prescribing would help my clients by … |  |
| Social prescribing would help my community by … |  |

TASK 2: VOTING

Scan QR code, or go to XXX to vote on the draft social prescribing model


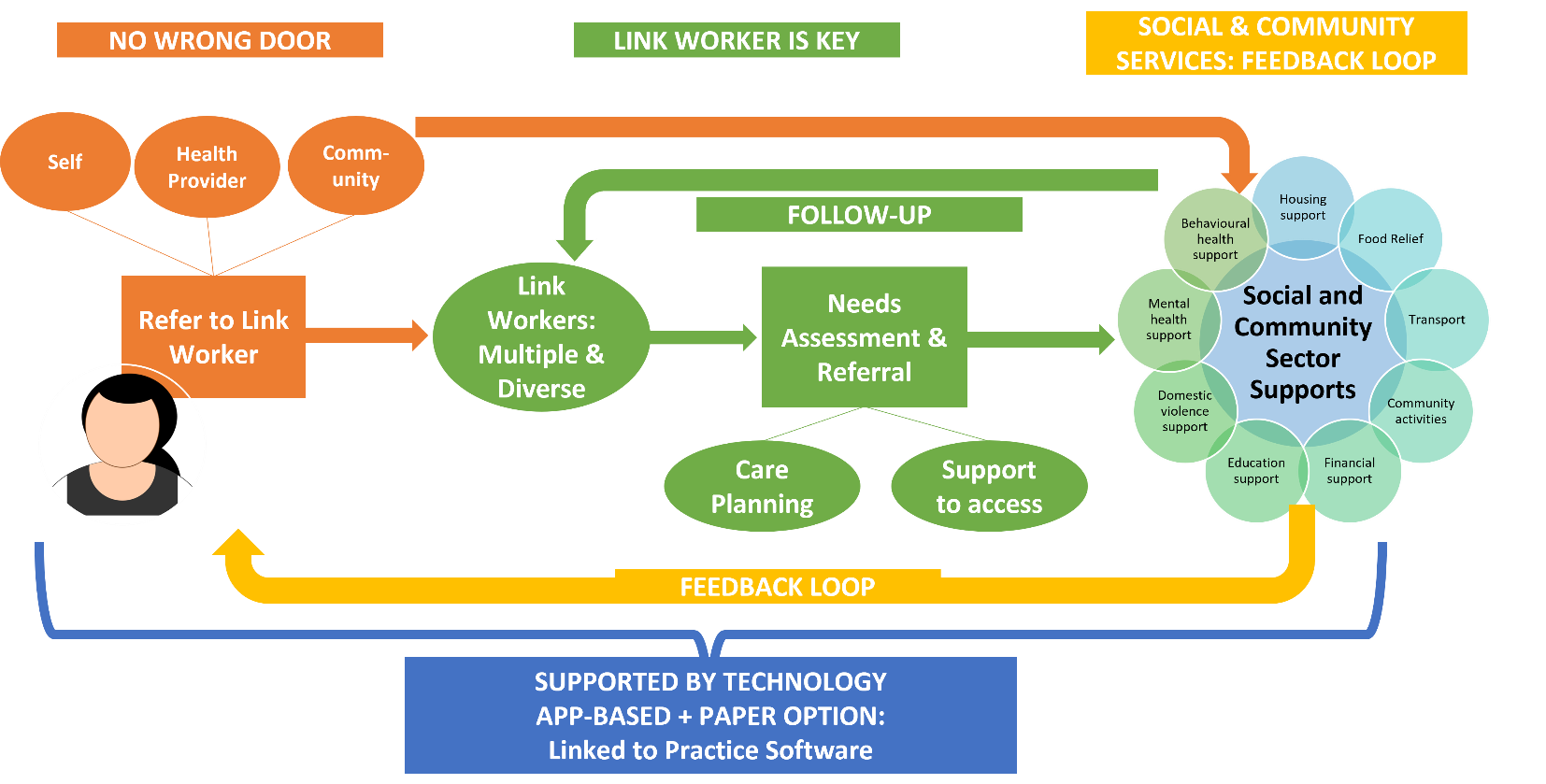


**QR CODE HERE**

TASK 3: DEMOGRAPHICS

(For statistical purposes only. Please write response or circle appropriate answer)

1. What is your profession?
2. About how long have you worked in your profession? A. 1 year or less B. 2-5 years C. >5 years
3. What is your gender? A. Male B. Female C. Non-binary D. Prefer not to answer
4. Which age group do you belong to?
5. Under 25yo B. 26-35yo C.36-45yo D.46-55yo E.56-65yo F.65+yo G. Prefer not to answer

Thank you for completing these questions. We will shortly return to the group discussion.

**SOCIAL PRESCRIBING CO-DESIGN**

**SERVICE PROVIDER WORKSHOP 2**

**SMALL GROUP ACTIVITY**

**NO WRONG DOOR**

**Anyone can refer to a Link Worker**

(Health Provider, Self-referral, Community referral)

| **Refer to Link Worker** | **THINK ABOUT:** | **PROGRAM ENTRY**  **How do service providers determine who might benefit from social prescribing?** (e.g., professional judgement, routine screening)  **How do individuals and community find out about social prescribing?** (e.g., print/social media advertising, flyers in health and community settings)  **LINK WORKER REFERRAL**  **How do service providers refer individuals to a Link Worker?** (e.g., warm referral, referral pad, online)  **How do individuals/family/community refer individuals to a Link Worker?** (e.g., warm referral, online) |
| --- | --- | --- |

**LINK WORKER IS KEY: WHO SHOULD THEY BE?**

**There are multiple Link Worker roles** (‘Professional’ and volunteer)

|  | **THINK ABOUT:** | **LINK WORKER SKILLS**  **What skills does a Link Worker need?** (e.g., communication skills, supporting clients with their mental health during social prescribing, self-care as a Link Worker)  **USE OF EXISTING AND NEW LINK WORKER ROLES**  **Existing Link Worker roles?** (e.g., Community Connections Program for adults aged 18-64; Care Finder for older adults eligible for aged care services; NDIS)  **New Link Worker roles?** (e.g., ‘Professional’ (paid) position; Volunteer positions; expanding existing roles e.g., Practice Nurse)  **LINK WORKER SUPERVISION**  **How are Link Workers supervised and supported?** (e.g., new supervision role, existing council staff for volunteers, community of practice) |
| --- | --- | --- |

**LINK WORKER IS KEY: WHAT DO THEY DO?**

| **Needs Assessment & Referral**  **FOLLOW-UP** | **THINK ABOUT:** | **NEEDS ASSESSMENT**  **How are the client’s needs determined by the Link Worker?** (e.g., through a conversation, social needs screening tool)  **REFERRAL**  **How are clients referred to supports and services to address their needs?** (e.g., contacting the service with/for the client; take the client to the services; referral form and client contacts the service)  **CARE PLANNING**  **How are clients supported and followed up by the Link Worker?** (e.g., case management with regular appointments; check-in phone calls) |
| --- | --- | --- |

**FEEDBACK LOOP**

| **FEEDBACK LOOP** | **THINK ABOUT:** | **WHO?**  **Who is provided feedback?**  **WHAT?**  **What information are they provided?**  **WHEN?**  **When does this happen?** (e.g., once at the end of a period of time, at regular intervals)  **HOW?**  **How is feedback provided?**  **How is a client’s privacy protected?** |
| --- | --- | --- |

**SUPPORTED BY TECHNOLOGY**

| **SUPPORTED BY TECHNOLOGY**  **APP-BASED + PAPER OPTION:**  **Linked to Practice Software** | **THINK ABOUT:** | **DIRECTORY OF SERVICS**  **What area does it cover** (e.g., local, state-wide, national)  **How is the directory maintained?** (e.g., locally maintained by each area, state-wide, national)  **What information is included about the services?** (e.g., patient eligibility, fees, wait times/urgent appointments, location, client feedback rating)  **REFERRAL & FEEDBACK LOOP**  **Inclusion of a social needs screening tool?**  **Linked to/integrated with existing software?**  **CARE PLANNING**  **Case management for social prescribers?**  **ASSESSMENT (BEFORE/AFTER ASSESSMENT TOOL)?**  **PATIENT PORTAL** |
| --- | --- | --- |

**SOCIAL PRESCRIBING CO-DESIGN**

**COMMUNITY WORKSHOP**

**SMALL GROUP ACTIVITY: CASE STUDIES AND JOURNEY MAPS**

**NOTE**: Images are stock images from Microsoft Word and do not represent real cases. Images are masked as per journal guidelines.


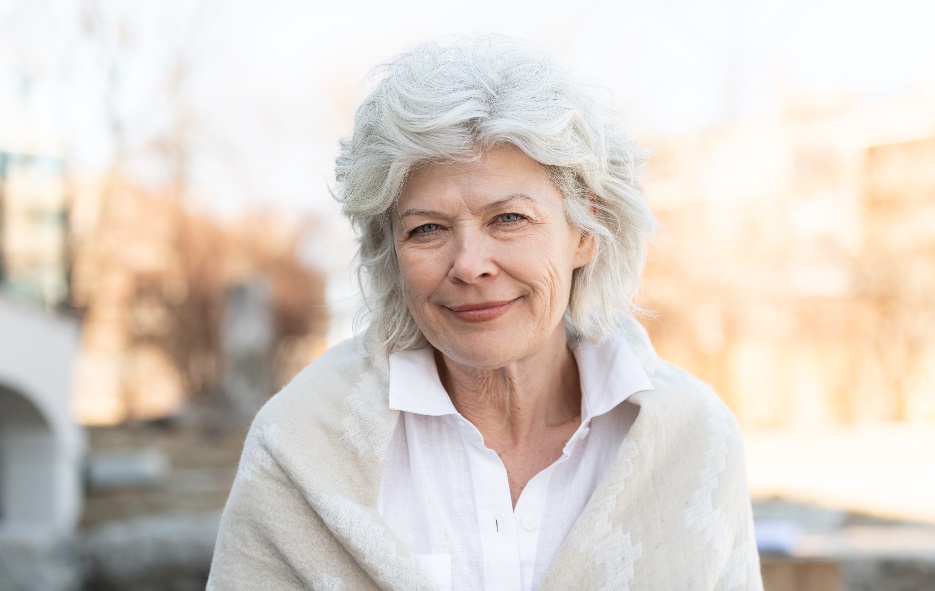


**Name**: Anna

**Age**: 78

**Marital status**: Recently widowed

**Family**: Son and grandchildren live in Queensland

**Occupation**: Retired interior designer

“I’d love to see more of my grandchildren. They are growing up so fast.”

**SOCIAL PRESCRIBING: ALLIED HEALTH REFERRAL PATHWAY**

Anna lives alone since her husband passed away last year. She misses her family, who live in Queensland. Anna’s son has been trying to persuade her to communicate with her grandchildren using the iPad he bought her when he last visited, but she is nervous to use the iPad.

Anna has been undergoing rehabilitation with a physiotherapist after breaking her hip when she fell while walking her dog. Anna has lost confidence being out and about since the fall. She finds it difficult to shop and cook for one person, so she tends to eat toast and canned salmon for dinner.

Anna’s physiotherapist is happy with her progress and suggested she no longer needs to attend the clinic. But Anna keeps coming back because she is feeling lonely and enjoys the company.

**Social needs**: Social connection, assistance with shopping and preparing meals, technology skills

| **How does the Physiotherapist know Anna needs extra support?** | **How does the Physiotherapist contact the Link Worker?** | **What happens in the appointment with the Link Worker?** | **How does the Link Worker connect Anna with social connection and other supports?** | **How does the Link Worker follow up with Anna to see how she’s doing?** | **How does the Link Worker let the Physiotherapist know how Anna is doing?** |
| --- | --- | --- | --- | --- | --- |
| - Conversation - Screening tool (during appointment/ in waiting room/ when doing an online booking) | - Phone call - Written referral - Online referral | Where should the link worker be located?  What should the link worker do?   - Screen for social needs - Have a conversation - Develop a support plan | Link worker:   - Phone call - Written referral - Online referral - Go to the appointment/ community group with Anna   If the Physiotherapist wants to do this without the Link Worker, how could they find out what services are available? | How often should the Link Worker follow up with Anna to see how she’s doing?  How should the follow- up be done (in person, phone, etc.)? | Who else should know about how Anna is doing?  How much information should be shared?   - Basic (e.g., I have received the referral; I have met with Anna) - Detailed (e.g., I have connected Anna to these supports; this is how he is doing)   How should this information be shared?   - Phone call - In writing - Online / App |


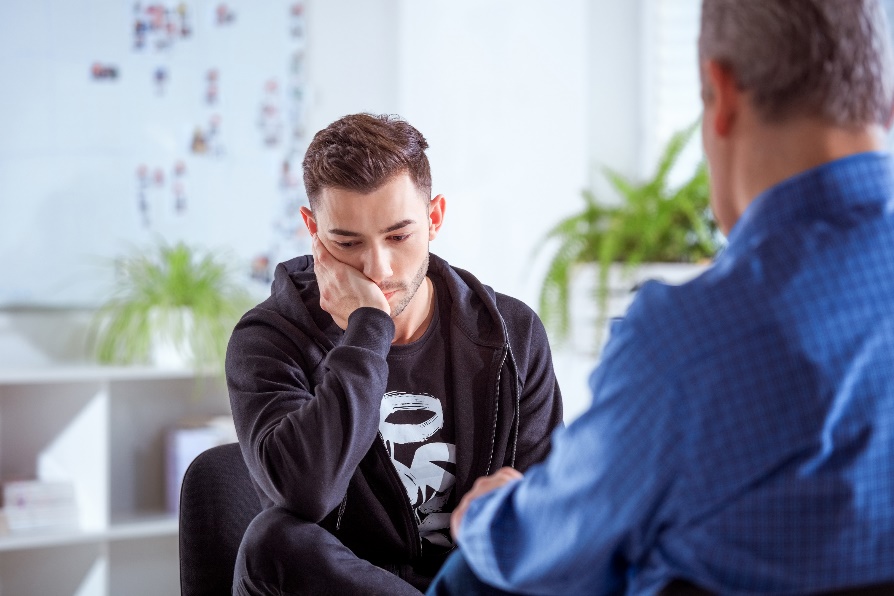


**Name**: Cooper

**Age**: 19

**Marital status**: Single

**Family**: Lives at home with his parents

**Occupation**: Unemployed

“I love working with computers, but I don’t know how to turn this into meaningful employment.”

**SOCIAL PRESCRIBING: GP REFERRAL PATHWAY**

Cooper finished school a year ago. He is unemployed and spends a lot of time at home playing computer games. Cooper and his dad fight a lot about how he spends his time. Cooper tends to eat snack food rather than meals and has poor sleeping habits.

His mum is worried about his mental health and makes an appointment for him to see his GP for a mental health assessment.

**Social needs**: Employment/education, social connection, family issues

| **How does the GP know Cooper needs extra support?** | **How does the GP contact the Link Worker?** | **What happens in the appointment with the Link Worker?** | **How does the Link Worker connect Cooper with job support and social connections?** | **How does the Link Worker follow up with Cooper to see how he’s doing?** | **How does the Link Worker let the GP know how Cooper is doing?** |
| --- | --- | --- | --- | --- | --- |
| - Conversation - Screening tool (during appointment/ in waiting room/ when doing an online booking) | - Phone call - Written referral - Online referral | Where should the link worker be located?  What should the link worker do?   - Screen for social needs - Have a conversation - Develop a support plan | Link worker:   - Phone call - Written referral - Online referral - Go to the appointment/ community group with Cooper   If the GP wants to do this without the Link Worker, how could they find out what services are available? | How often should the Link Worker follow up with Cooper to see how he’s doing?  How should the follow- up be done (in person, phone, etc.)? | Who else should know about how Cooper is doing?  How much information should be shared?   - Basic (e.g., I have received the referral; I have met with Cooper) - Detailed (e.g., I have connected Cooper to these supports; this is how he is doing)   How should this information be shared?   - Phone call - In writing - Online / App |


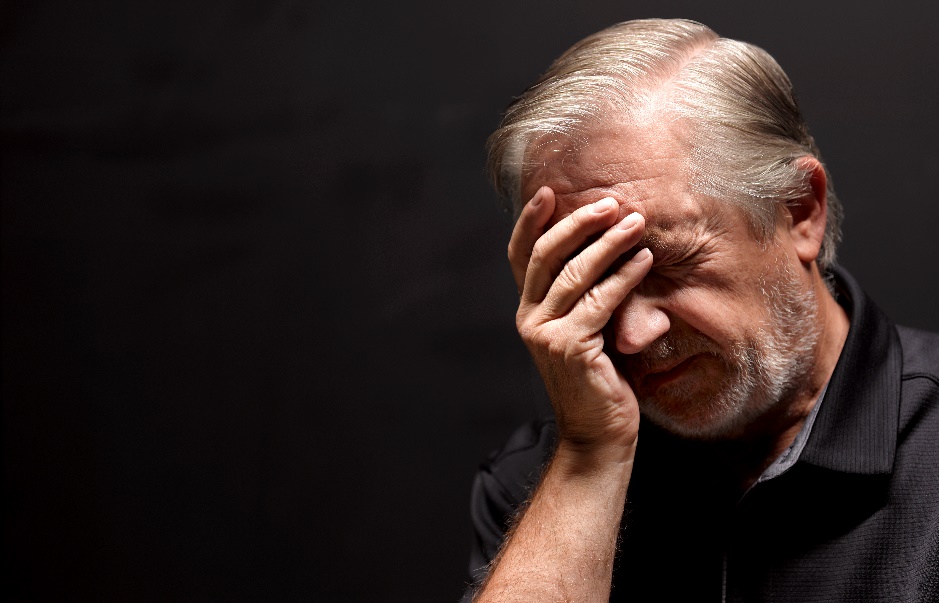


**Name**: Jim

**Age**: 68

**Marital status**: Married

**Family**: Adult children living in Adelaide

**Occupation**: Retired software engineer

“One of the best things about my job was the sense of mateship with my co-workers. I really miss that now I’m retired.”

**SOCIAL PRESCRIBING: GP REFERRAL PATHWAY**

Jim and his wife, Deb, were looking forward to their retirement. They bought a caravan and planned many trips across Australia. Unfortunately, Deb had a stroke 18-months ago and has difficulties walking, toileting, and feeding herself. Jim spends his time caring for Deb.

Jim regularly sees his GP and has a Chronic Disease Management plan for his diabetes.

Jim is feeling low and isolated and feels like he is “stuck in a rut” and unsure how to move forward with his life.

**Social needs**: Carer support, social connection

| **How does the GP know Jim needs extra support?** | **How does the GP contact the Link Worker?** | **What happens in the appointment with the Link Worker?** | **How does the Link Worker connect Jim with carer support and social connections?** | **How does the Link Worker follow up with Jim to see how he’s doing?** | **How does the Link Worker let the GP know how Jim is doing?** |
| --- | --- | --- | --- | --- | --- |
| - Conversation - Screening tool (during appointment/ in waiting room/ when doing an online booking) | - Phone call - Written referral - Online referral | Where should the link worker be located?  What should the link worker do?   - Screen for social needs - Have a conversation - Develop a support plan | Link worker:   - Phone call - Written referral - Online referral - Go to the appointment/ community group with Jim   If the GP wants to do this without the Link Worker, how could they find out what services are available? | How often should the Link Worker follow up with Jim to see how he’s doing?  How should the follow- up be done (in person, phone, etc.)? | Who else should know about how Jim is doing?  How much information should be shared?   - Basic (e.g., I have received the referral; I have met with Jim) - Detailed (e.g., I have connected Jim to these supports; this is how he is doing)   How should this information be shared?   - Phone call - In writing - Online / App |


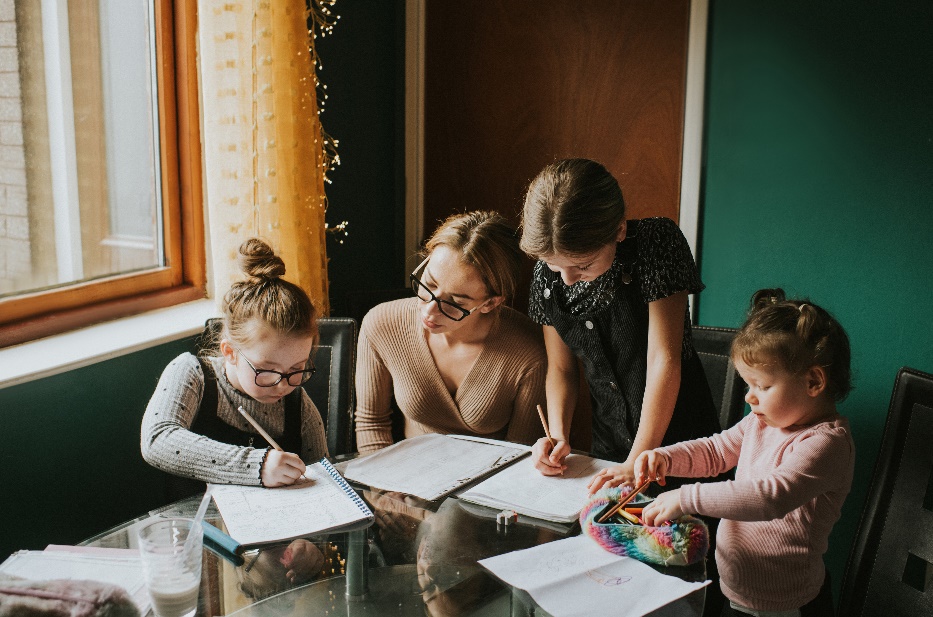


**Name**: Marianne

**Age**: 33

**Marital status**: Divorced

**Family**: Parents and siblings live in Holland, where Marianne was born

**Occupation**: Works part-time as a house cleaner

“The most important thing to me are my children. I just want to be there for them and be the best mum I can be.”

**SOCIAL PRESCRIBING: SELF/COMMUNITY REFERRAL PATHWAY**

Marrianne is a single mother with three children. She works part time as a house cleaner while her children are at kindy and school. Her family live overseas.

Marianne works hard to support her family but she’s finding it harder to get by because of recent changes to her Family Tax Benefit income. With rising food prices, Marianne recently found herself in a situation where she couldn’t afford to buy food for her family.

Marianne is feeling very stressed and worried about what people will think of her. In desperation, she reached out to the local community through the Community Facebook page to ask if anyone could help her out with some food. Her community was able to help with her immediate food needs but were not aware of how to connect her with longer term support.

**Social needs**: Financial assistance, food relief, financial counselling

| **How does Marianne find out about the social prescribing program?** | **How does Marianne contact the Link Worker?** | **What happens in the appointment with the Link Worker?** | **How does the Link Worker connect Marianne with food relief and financial counselling?** | **How does the Link Worker follow up with Marianne to see how she’s doing?** | **How could Marianne find out about food relief and financial counselling herself if she doesn’t want to see a Link Worker?** |
| --- | --- | --- | --- | --- | --- |
| - Printed flyers in the community - Printed flyers in health settings (GP, pharmacy) - Local paper - Social media | - Phone call - Online form | Where should the link worker be located?  What should the link worker do?   - Screen for social needs - Have a conversation - Develop a support plan | Link worker:   - Phone call - Written referral - Online referral - Go to the service/ community group with Marianne | How often should the Link Worker follow up with Marianne to see how she’s doing?  How should the follow- up be done (in person, phone, etc.)? | - Printed booklet - Online service directory - Social media |


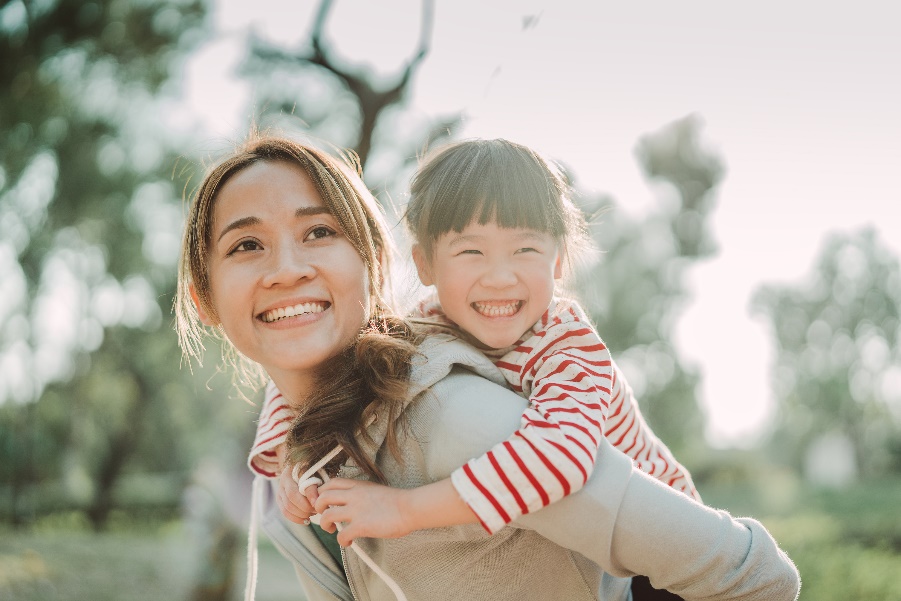


**Name**: Paula

**Age**: 29

**Marital status**: De-facto relationship

**Family**: Parents and siblings live in Adelaide

**Occupation**: Stay-at-home mum

“I’ve been feeling really lonely since moving here. I’d love to meet some people and feel part of the community.”

**SOCIAL PRESCRIBING: ALLIED HEALTH REFERRAL PATHWAY**

Paula recently moved to the Barossa with her partner and her 5-year-old daughter. Paula is a stay-at-home mum and doesn’t have any friends in the local community.

Paula’s daughter has speech and communication issues and sees a speech pathologist.

Paula is feeling socially isolated and is finding it difficult to find friends and social connection in the community.

**Social needs**: Social connection

| **How does the Speech Pathologist know Paula needs extra support?** | **How does the Speech Pathologist contact the Link Worker?** | **What happens in the appointment with the Link Worker?** | **How does the Link Worker connect Paula with social connections?** | **How does the Link Worker follow up with Paula to see how she’s doing?** | **How could Paula find out about available services and supports herself if she doesn’t want to see a Link Worker?** |
| --- | --- | --- | --- | --- | --- |
| - Conversation - Screening tool (during appointment/ in waiting room/ when doing an online booking) | - Phone call - Written referral - Online referral | Where should the link worker be located?  What should the link worker do?   - Screen for social needs - Have a conversation - Develop a support plan | Link worker:   - Phone call - Written referral - Online referral - Go to the appointment/ community group with Paula   If the Speech Pathologists wants to do this without the Link Worker, how could they find out what services are available? | How often should the Link Worker follow up with Paula to see how she’s doing?  How should the follow- up be done (in person, phone, etc.)? | Who else should know about how Paula is doing?  How much information should be shared?   - Basic (e.g., I have received the referral; I have met with Paula) - Detailed (e.g., I have connected Paula to these supports; this is how he is doing)   How should this information be shared?   - Phone call - In writing - Online / App |
